# Dynamic Acetylation of All Lysine 4–Methylated Histone H3 in the Mouse Nucleus: Analysis at c-*fos* and c-*jun*

**DOI:** 10.1371/journal.pbio.0030393

**Published:** 2005-11-08

**Authors:** Catherine A Hazzalin, Louis C Mahadevan

**Affiliations:** **1**Nuclear Signalling Laboratory, Department of Biochemistry, University of Oxford, Oxford, United Kingdom; Adolf Butenandt InstituteGermany

## Abstract

A major focus of current research into gene induction relates to chromatin and nucleosomal regulation, especially the significance of multiple histone modifications such as phosphorylation, acetylation, and methylation during this process. We have discovered a novel physiological characteristic of all lysine 4 (K4)–methylated histone H3 in the mouse nucleus, distinguishing it from lysine 9–methylated H3. K4-methylated histone H3 is subject to continuous dynamic turnover of acetylation, whereas lysine 9–methylated H3 is not. We have previously reported dynamic histone H3 phosphorylation and acetylation as a key characteristic of the inducible proto-oncogenes c-*fos* and c-*jun.* We show here that dynamically acetylated histone H3 at these genes is also K4-methylated. Although all three modifications are proven to co-exist on the same nucleosome at these genes, phosphorylation and acetylation appear transiently during gene induction, whereas K4 methylation remains detectable throughout this process. Finally, we address the functional significance of the turnover of histone acetylation on the process of gene induction. We find that inhibition of turnover, despite causing enhanced histone acetylation at these genes, produces immediate inhibition of gene induction. These data show that all K4-methylated histone H3 is subject to the continuous action of HATs and HDACs, and indicates that at c-*fos* and c-*jun,* contrary to the predominant model, turnover and not stably enhanced acetylation is relevant for efficient gene induction.

## Abstract 2

Continuous turnover rather than stable acetylation of histone H3 (methylated at Lysine 4) is necessary for the induction of certain genes including c-fos and c-jun.

## Introduction

**Figure 1:**
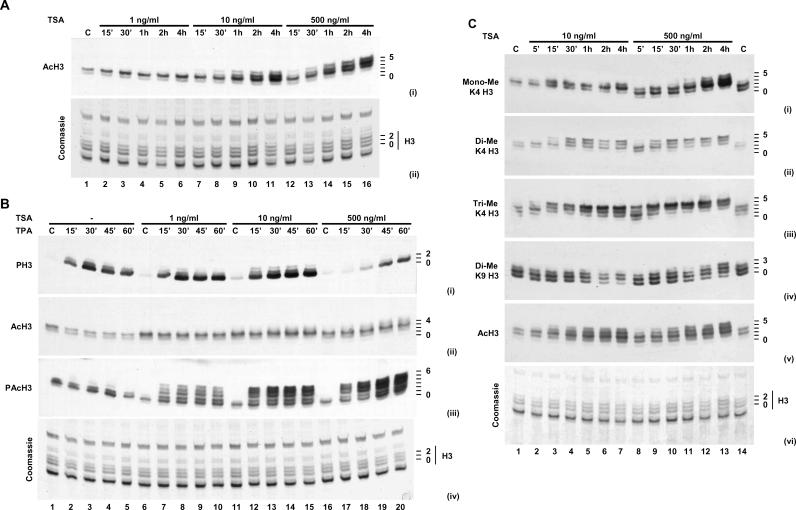
Acetylation and Methylation of Histone H3 TSA- and TPA-Treated Cells (A) Quiescent C3H 10T½ cells were treated with increasing concentrations of TSA (1, 10, or 500 ng/ml; 15 min to 4 h). “C” indicates control (unstimulated). (B) Quiescent C3H 10T½ cells were untreated (−) or pre-treated with increasing concentrations of TSA (1, 10, or 500 ng/ml; 15 min). Cells were left unstimulated (C) or stimulated with TPA (15 to 60 min). (C) Quiescent C3H 10T½ cells were treated with TSA (10 or 500 ng/ml; 5 min to 4 h). Acid-soluble proteins were extracted and separated on acid-urea gels. Western blots were carried out with anti-acetyl-H3 ([A], panel i; [B], panel ii; [C], panel v), anti-phospho-H3 ([B], panel i), anti-phosphoacetyl-H3 ([B], panel iii), anti-monomethyl-K4 H3 ([C], panel i), anti-dimethyl-K4 H3 ([C], panel ii), anti-trimethyl-K4 H3 ([C], panel iii), or anti-dimethyl-K9 H3 ([C], panel iv) antibodies. An equivalent gel was stained with Coomassie to control for protein loading ([A], panel ii; [B], panel iv; [C], panel vi). Positions of histone isoforms are shown on the right of each panel, with zero being unmodified histone H3.

Histone modifications have been co-located to specific genes by chromatin immunoprecipitation (ChIP) assays or by immunocytochemistry, and flowing from that, their functions in processes involving these genes, such as epigenetic cellular memory, silencing, and transcriptional regulation, have been implied (reviewed in [1,2]). However, the extraordinary biochemical susceptibility of histone tails carrying one modification to further modification has received little attention. The first clear example of such biochemical compartmentalisation in the mouse nucleus was the observation that all histone H3 phosphorylated at serine 10 (S10) becomes immediately and very highly acetylated upon treatment with histone deacetylase (HDAC) inhibitors sodium butyrate [3] or Trichostatin A (TSA) [4]. This was revealed by analysis of the modification state of ^32^P-radiolabelled H3 on acid-urea gels, in which each additional acetylation or phosphorylation event causes an incremental shift, giving rise to a “ladder” of increasingly modified H3 bands (see Figure 1). Two aspects of this observation deserve emphasis. First, the majority of Coomassie-stainable H3 is resistant to TSA treatment, remaining in lower rungs of the H3 ladder on these gels. Second, by contrast, phosphorylated H3 responds not only quantitatively and especially sensitively to such treatment, but rises to occupy the highest possible rungs of the H3 ladder, indicating that on phosphorylated H3, most, if not all, available lysines in the H3 tail become acetylated. This shows that in mouse nuclei, blockade of HDACs results in histone acetyltransferases (HATs) extensively modifying all available lysines on a tiny fraction of phosphorylated H3 tails rather than random lysine residues on all tails throughout the nucleus.

The availability of modification-specific antibodies for histones H3 and H4 allowed use of ChIP assays to identify specific genes that showed the TSA-responsive trait of continuous dynamic acetylation. Since c-*fos* and c-*jun* nucleosomes carried phosphoacetylated histone H3 upon gene activation [4], these genes were tested and shown to become hyperacetylated upon TSA treatment [5]. These studies showed also that c-*fos* and c-*jun* nucleosomes became hyperacetylated even when cells were not stimulated, when these genes were inactive and not therefore carrying any phosphorylated H3. This implied that HATs and HDACs are constitutively targeted to these genes, causing continuous turnover of acetylation in unstimulated cells. Further, TSA sensitivity of phosphorylated H3 might simply be a reflection of the fact that phosphorylation is also targeted to these same cycling nucleosomes upon stimulation of these cells.

In this paper, we first extend characterisation of dynamic acetylation in the mouse nucleus by analysis of H3 methylation. Histone H3 can be methylated at lysine 4 (K4) and/or lysine 9 (K9), the former being generally associated with active or poised genes [6–8] and the latter with repressed genes [9,10], although it is now emerging that both modifications can co-exist on the same genes ([11]; reviewed in [2]). We show that all K4-methylated H3 is also subject to dynamic acetylation, whereas K9-methylated H3 is not, a clear and unambiguous physiological difference between these two modifications. Further, we show that c-*fos* and c-*jun* nucleosomes are highly methylated at K4 irrespective of whether these genes are induced or not. Upon gene activation, acetylation and phosphorylation co-exist transiently on K4-methylated histone H3 tails at c-*fos* and c-*jun*. Secondly, we have analysed the consequence of HDAC blockade and hyperacetylation of these nucleosomes on the process of c-*fos* and c-*jun* induction. Contrary to expectation, this analysis showed that elevated acetylation does not correspond to enhanced expression of these genes, but in fact, inhibits their induction. This argues against the predominant model, whereby enhanced histone acetylation at these genes correlates with transcription, and suggests instead that it is turnover of histone acetylation that is relevant.

## Results

### Dynamic Acetylation of K4-Methylated Histone H3 in the Mouse Nucleus

Titration experiments were first carried out to biochemically define TSA-hypersensitive chromatin (Figure 1A). Histones from cells treated with TSA at 1, 10, and 500 ng/ml over a 4-h time course were analysed on acid-urea gels on which each additional acetyl modification causes an incremental shift of H3 up the ladder. As reported previously [4,5], the Coomassie-stained gel (Figure 1A, panel ii) showed very little effect on bulk H3 even at high concentrations and long TSA treatments. However, Western blotting with anti-acetyl-H3 antibody (Figure 1A, panel i) clearly showed increased acetyl-H3 at higher positions on the H3 ladder above the major stainable H3 bands, indicating that a minute fraction of H3 becomes fully modified. Compared to control cells, this increase in intensity and appearance of higher acetylated H3 bands could be seen at 10 ng/ml TSA and 15–30 min time points.

Due to occlusion by phosphate on S10, this anti-acetyl-H3 antibody only recognises H3 that is not phosphorylated (for data, see Figure 1 of [5]; A. L. Clayton, L. C. M., unpublished data). We also investigated early TSA-sensitive events (Figure 1B) using our (acetyl-K9/phospho-S10) phosphoacetyl antibody (characterised in [4]). At concentrations as low as 1 ng/ml TSA and at the earliest time point tested, there was extensive appearance of phosphoacetyl-H3 at the highest positions on the H3 ladder (Figure 1B, panel iii, lane 7). This confirms previous studies discussed above that all phosphorylated H3 is hypersensitive to TSA-induced acetylation, with a fraction rapidly appearing in fully acetylated form, despite bulk Coomassie-stained H3 being largely unaffected (Figure 1B, panel iv). For all further work, we used 10 ng/ml TSA at short time courses as definitive of TSA hypersensitivity, although effects can clearly be seen at 1 ng/ml.

In this study, we analysed other H3 modifications for the TSA-hypersensitive trait and found that H3 methylated at K4 falls in this category (Figure 1C). Note that methylation does not cause any shift of histone H3 on acid-urea gels. TSA treatment at 10–500 ng/ml resulted in rapid acetylation of K4 that was mono- (Figure 1C, panel i), di- (panel ii), and tri-methylated (panel iii), as evidenced by progress up the H3 ladders. This effect was particularly clear with trimethylated K4 (trimethyl-K4) H3, where appearance of higher bands corresponded with complete loss of lower bands of the ladder (Figure 1C, panel iii). By contrast, H3 dimethylated at K9 was resistant to TSA. Although a small effect could be seen at higher concentrations and later time points (Figure 1C, panel iv), this largely correlated with the slight effect of TSA on bulk H3, as seen in Coomassie-staining H3 bands (panel vi).

Over many similar experiments, we conclude that in the mouse nucleus, a defining characteristic of virtually all histone H3 methylated on K4, particularly trimethyl-K4 H3, is its TSA hypersensitivity. This indicates that most or all of histone H3 methylated at K4 in the mouse nucleus is targeted for continuous acetylation and deacetylation, such that inhibition of the latter by TSA produces immediate and extensive acetylation, exactly as described for phospho-S10 H3. Although previous analyses have localised K4- and K9-methylated H3 at different genes, alluding to their differing functions, this is the first description to our knowledge of a clear physiological distinction between histone H3 methylated at these two residues.

### TSA Hypersensitivity Is Targeted to Nucleosomes at c-*fos* and c-*jun*

**Figure 2:**
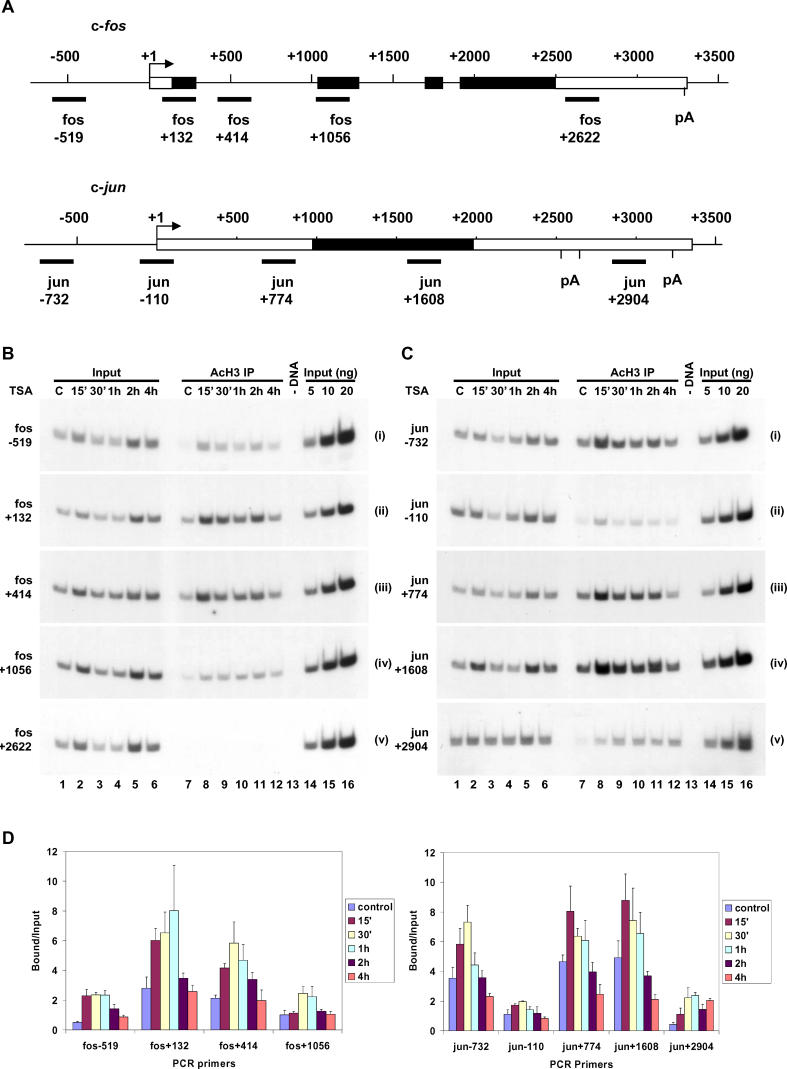
Effect of TSA Treatment on Acetylation of Nucleosomes Associated with c-*fos* and c-*jun* Genes (A) Schematic diagram representing relative positions of regions of c-*fos* and c-*jun* amplified by primer pairs used in the PCR step of ChIP assays. Exons are indicated by boxes, open for untranslated regions and filled for coding regions. Polyadenylation sites (pA) are indicated. (B and C) Cross-linked chromatin fragments were prepared from quiescent C3H 10T½ cells treated with TSA (10 ng/ml; 15 min to 4 h). “C” indicates control (unstimulated). Specific DNAs were immunoprecipitated with anti-acetyl-H3 antibodies. Recovered DNAs from antibody-bound fractions (AcH3 IP) as well as total input DNA (Input) from released chromatin used for ChIP were analysed for the presence of c-*fos* (B) and c-*jun* (C) gene sequences. Controls for PCR included a DNA minus (−DNA) reaction and 5- to 20-ng loadings of input DNA to ensure all amplifications were within the linear range. PCR reactions were carried out in triplicate and gels quantified by phosphorimaging. Representative gels are shown. (D) Data are expressed graphically as average Bound/Input (± standard deviation). Note that to correct for the dilution factor applied to anti-acetyl-H3 immunoprecipitated DNA prior to PCR analyses, values obtained from quantification of PCR gels were multiplied by three.

Previous studies have indicated that histones at c-*fos* and c-*jun* chromatin are rapidly acetylated in the presence of high concentrations of TSA [5]. We next used ChIP assays to ask if TSA hypersensitivity could be observed at specific regions of c-*fos* and c-*jun,* shown schematically in Figure 2A. Cells treated with TSA (10 ng/ml) for 15 min to 4 h were assayed by ChIP with anti-acetyl-H3 antibody. PCR was used to probe immunoprecipitated DNA for specific regions of c-*fos* (Figure 2B, panels i–v) and c-*jun* (Figure 2C, panels i–v). TSA treatment (10 ng/ml) clearly produced targeted acetylation at these genes that was detectable, and in many cases maximal, at the earliest time point tested (15 min), and then dropped off to almost basal levels by 4 h (Figure 2B–2D). For both genes, TSA-enhanced acetylation was region-specific. For c-*fos,* +132 and +414, representing the first exon and intron, respectively, showed the clearest enhancement, whereas regions that flank this, i.e., the promoter, −519, and second exon, +1056, showed a weaker response. No increase in acetylation was detectable further downstream at +2622 (Figure 2B). For c-*jun,* apart from the region around the transcription start site (−110), which showed a poor response as reported previously [5], and +2904 at the 3′ end of the gene, the promoter (−732) and other coding regions (+774 and +1608) showed very clear responses to TSA (Figure 2C). Note that Bound/Input values for ChIP assays plotted here (Figure 2D) are a measure of total quantitative enhancement at each position. However, because of lower background acetyl-H3 signal at some positions in control cells, the fold-stimulation values between control and stimulated cells (Figure S1) appear greatest where this background is lowest.

Because TSA does not induce these genes (see below), these effects are unrelated to transcription. Furthermore, there was a clear correlation between pre-existing levels of acetylation at each region in control cells and its sensitivity to TSA treatment. Regions with no pre-existing acetylation (*fos* +2622) showed no TSA enhancement, whereas those with low (*fos* −519 and +1056, and *jun* −110 and +2904) and higher (*fos* +132 and +414, and *jun* −732, +774, and +1608) pre-existing levels showed TSA enhancement to intermediate and high levels, respectively. The basal level was presumably a reflection of continuous turnover at these positions. Finally, whenever detected, TSA-enhanced acetylation was close to maximal at early time points (15–30 min), dropping off to return to almost basal levels by 4 h (Figure 2D), in striking contrast to acetylation in the nucleus as a whole (see Figure 1A, panel i, lanes 7–11), which rises very strongly throughout this period. Localised reversal of acetylation is therefore highly gene-specific and must be mediated either by TSA-insensitive HDACs or possibly by a histone H3 replacement mechanism.

### K4 Trimethylation of Histone H3 at c-*fos* and c-*jun*

**Figure 3:**
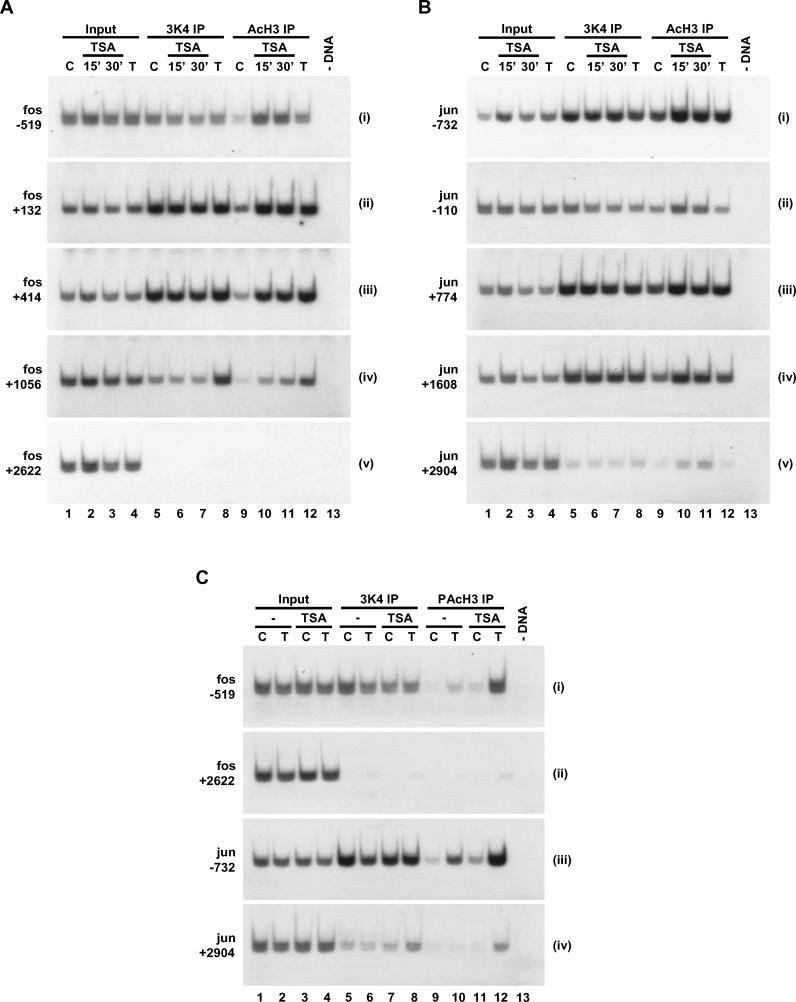
Histone H3 K4 Trimethylation and TSA Hypersensitivity at c-*fos* and c-*jun* (A and B) Cross-linked chromatin fragments were prepared from quiescent C3H 10T½ cells treated with TSA (10 ng/ml; 15 to 30 min) or stimulated with TPA (T; 30 min). “C” indicates control (unstimulated). Chromatin fragments were immunoprecipitated with anti-trimethyl-K4 H3 (3K4 IP) or anti-acetyl-H3 (AcH3 IP) antibodies. (C) Cross-linked chromatin fragments were prepared from quiescent C3H 10T½ cells untreated (−) or pre-treated with TSA (10 ng/ml; 15 min). Cells were left unstimulated (C) or stimulated with TPA (T; 30 min). Chromatin fragments were immunoprecipitated with anti-trimethyl-K4 H3 (3K4 IP) or anti-phosphoacetyl-H3 (PAcH3 IP) antibodies. DNA recovered from antibody-bound fractions as well as total input DNA (Input) from released chromatin used for ChIP was analysed for the presence of c-*fos* ([A]; [C], panels i and ii) and c-*jun* ([B]; [C], panels iii and iv) gene sequences. DNA immunoprecipitated with anti-acetyl-H3 antibody was diluted one in three, anti-trimethyl-K4 H3 was diluted one in 20, and anti-phosphoacetyl-H3 antibody was not diluted before PCR analyses.

From data in Figure 1C, TSA hypersensitivity of c-*fos* and c-*jun* nucleosomes implies that they may also carry trimethyl-K4 H3, verifiable by ChIP assays (Figure 3). Preliminary experiments showed that trimethyl-K4 H3 antibody recovered much more of these chromatin fragments than di- or monomethyl antibodies (data not shown). Direct comparison of regions of c-*fos* (Figure 3A) and c-*jun* (Figure 3B) recovered by anti-trimethyl-K4 and anti-acetyl antibodies showed remarkably that the distribution and relative levels of trimethyl-K4 and acetylated H3 parallel each other across these genes. Acetylation levels increased in response to TSA treatment, whereas trimethyl-K4 levels remained unaffected (Figure 3A and 3B, compare lanes 9–11 and 5–7). At c-*fos,* both modifications were high at +132 and +414 and intermediate at −519 and +1056, but both were undetectable at +2622 (Figure 3A). Similarly, for c-*jun,* both modifications were detectable at −732, +774, and +1608, but both fell to lower levels at −110 and +2904 (Figure 3B).

We also performed ChIP assays to examine histone H3 acetylation and phosphoacetylation at c-*fos* and c-*jun* in 12-O-tetradecanoyl phorbol-13-acetate (TPA)–stimulated cells. Like many other stimuli [5], TPA produced acetylation (Figure 3A and 3B, lanes 9 and 12) and phosphoacetylation at these genes, with higher levels of the latter detectable in TSA-treated cells (Figure 3C, lanes 9–12; data not shown). Unlike acetylation and phosphorylation, which are rapidly induced upon stimulation and gene induction, K4 H3 methylation remained detectable throughout this time course with all stimuli tested (Figure 3; data not shown). At all regions where it was detected, a clear K4 H3 methylation signal was seen in unstimulated cells, and upon TPA treatment alone or in combination with TSA (Figure 3C, lanes 5–8; data not shown). This indicates that K4 H3 methylation occurs at these genes in quiescent cells and upon stimulation, raising the possibility that it functions as a marker to target these regions for further modification.

### K4 Methylation of Histone H3 and TSA Hypersensitivity at Constitutively Active and Silent Genes

**Figure 4:**
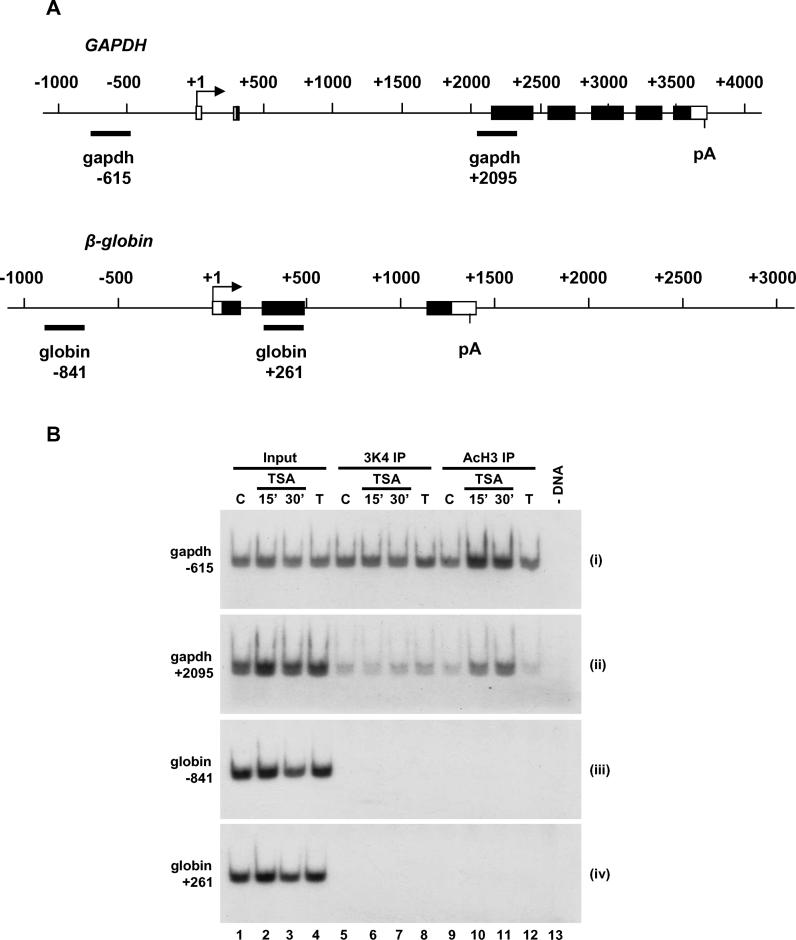
Histone H3 K4 Trimethylation and Acetylation at Other Genes (A) Schematic diagram representing relative positions of regions of *GAPDH* and β*-globin* amplified by primer pairs used in the PCR step of ChIP assays. Exons are indicated by boxes, open for untranslated regions and filled for coding regions. Polyadenylation sites (pA) are indicated. (B) Cross-linked chromatin fragments were prepared from quiescent C3H 10T½ cells treated with TSA (10 ng/ml; 15 to 30 min) or stimulated with TPA (T; 30 min). “C” indicates control (unstimulated). Specific DNAs were immunoprecipitated with anti-trimethyl-K4 H3 (3K4 IP) or anti-acetyl-H3 (AcH3 IP) antibodies. Recovered DNAs from antibody-bound fractions as well as total input DNA (Input) from released chromatin used for ChIP were analysed for the presence of *GAPDH* ([B], panels i and ii) and β*-globin* ([B], panels iii and iv) gene sequences. DNA immunoprecipitated with anti-acetyl-H3 antibody was diluted one in three and anti-trimethyl-K4 H3 was diluted one in 20 before PCR analyses.

We have now analysed many inducible genes (*fos*B, *jun*B, *jun*D, and *nur*77; data not shown) and found all have regions of K4 methylation and TSA hypersensitivity, the two traits being extremely well correlated. However, the literature shows that K4 methylation of histone H3 occurs at many active genes, whereas many repressed silent genes are not methylated at K4. We compared K4 trimethylation and TSA hypersensitivity at a constitutively expressed gene and a silent gene, *glyceraldehyde-3-phosphate dehydrogenase (GAPDH)* and β*-globin,* respectively, shown schematically in Figure 4A. The constitutively expressed *GAPDH* gene showed clear K4 methylation of histone H3 at −615 in the promoter and also in coding regions of the gene, with lower levels at position +2095. All regions analysed were subject to TSA-hypersensitive acetylation, maintaining the correlation between H3 K4 methylation and TSA hypersensitivity (Figure 4B, panels i and ii; data not shown). Despite these similarities, there was a clear difference: TPA stimulation, which activates c-*fos* and c-*jun* transcription but not *GAPDH,* enhanced acetylation at specific regions of c-*fos* and c-*jun* (Figure 3A and 3B, lanes 9 and 12), but had no effect at any position of *GAPDH* tested (Figure 4B, panels i and ii, lanes 9 and 12; data not shown). By contrast, the silent β*-globin* gene showed neither H3 K4 methylation nor TSA hypersensitivity nor any stimulus-dependent acetylation at any position analysed, including −840 and +261 (Figure 4B, panels iii and iv; data not shown).

These studies revealed three characteristic types of chromatin modification response in these cells. Inducible genes showed regions of H3 K4 methylation that were TSA-hypersensitive, and in addition, stimulus-dependent gene induction led to H3 acetylation and phosphoacetylation. The constitutively active *GAPDH* gene showed H3 K4 methylation and TSA hypersensitivity, but as might be expected, no stimulus-dependent acetylation. And finally, the silent β*-globin* gene showed none of these three characteristics. An important observation that emerges here is the correlation between K4 methylation and TSA hypersensitivity, which is as yet unbroken (see Discussion).

### Trimethyl-K4, Acetyl-K9, and Phospho-S10 Histone H3 Occur on the Same Nucleosome at c-*fos* and c-*jun*

Western blotting of H3 ladders on acid-urea gels (see Figure 1) showed that phospho-S10 H3 or trimethyl-K4 H3 shifted upwards upon TSA treatment, proving that acetyl groups must co-exist on the same H3 tails carrying these modifications. Further, the specificity of our phosphoacetyl antibody proves that acetyl-K9 and phospho-S10 must co-exist on the same H3 tail in stimulated cells. However, this leaves unanswered the question of whether trimethyl-K4 and phospho-S10 are themselves on the same nucleosome, or if they represent two separate populations of H3 that are independently TSA-hypersensitive.

**Figure 5:**
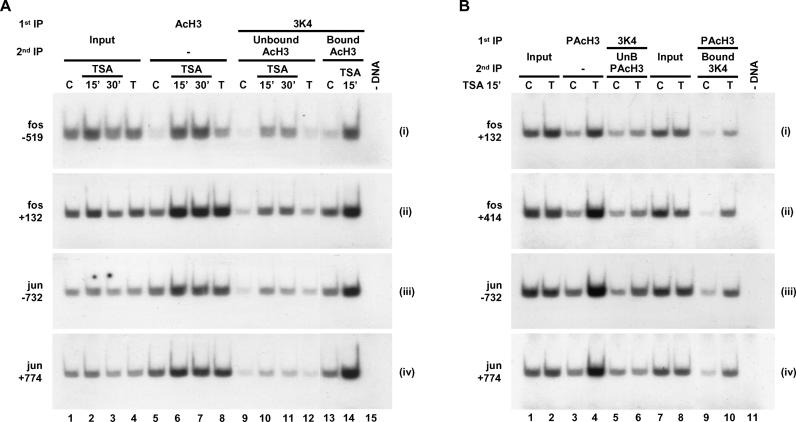
Three Histone H3 Modifications: Methylation, Acetylation, and Phosphorylation Are Targeted to the Same Nucleosome (A) Cross-linked chromatin fragments were prepared from quiescent C3H 10T½ cells treated with TSA (10 ng/ml; 15 to 30 min) or stimulated with TPA (T; 30 min). “C” indicates control (unstimulated). Specific DNAs were immunoprecipitated directly (1st IP; upper line) with anti-acetyl-H3 (AcH3) or anti-trimethyl-K4 H3 (3K4) before a second IP with anti-acetyl-H3 (2nd IP; lower line) to analyse chromatin in the anti-trimethyl-K4 H3 unbound or bound fraction (Unbound AcH3 and Bound AcH3, respectively). DNA immunoprecipitated with anti-acetyl-H3 antibody (1st or 2nd IP) was diluted one in three before PCR analyses. (B) Cross-linked chromatin fragments were prepared from quiescent C3H 10T½ cells pre-treated with TSA (10 ng/ml; 15 min). Cells were unstimulated (C) or stimulated with TPA (T; 30 min). Specific DNAs were immunoprecipitated directly (1st IP; upper line) with anti-phosphoacetyl-H3 (PAcH3) or anti-trimethyl-K4 H3 (3K4) before a second IP (2nd IP; lower line) to analyse chromatin in the unbound or bound fraction with anti-phosphoacetyl-H3 (Unbound PAcH3), or with anti-trimethyl-K4 H3 antibodies (Bound 3K4). DNA immunoprecipitated with anti-phosphoacetyl-H3 (1st or 2nd IP) was not diluted before PCR analyses. Recovered DNAs as well as total input DNA (Input) from released chromatin used for ChIP were analysed for the presence of c-*fos* ([A], panels i and ii; [B], panels i and ii) and c-*jun* ([A], panels iii and iv; [B], panels iii and iv) gene sequences.

In the absence of antibodies that recognise the triple modification, two approaches involving sequential immunoprecipitation (IP) were used to ask if all three modifications occur on the same nucleosomes at c-*fos* and c-*jun*. First, we asked if an antibody against one H3 modification (Figure 5, “1st IP”) would also recover H3 with a second modification, depletion of the co-immunoprecipitated epitope in the unbound fraction being verifiable by re-IP with a second antibody (Figure 5, “2nd IP Unbound”). Conversely, antibody-bound chromatin from a first IP was subject to a second round of IP with a different modification-specific antibody (Figure 5, “2nd IP Bound”). Positions along c-*fos* and c-*jun* analysed here correspond to peaks of modification seen in previous figures.

To investigate co-existence of trimethyl-K4 on acetylated H3 tails at c-*fos* and c-*jun,* chromatin from control and TSA-treated cells were first immunoprecipitated with trimethyl-K4 antibodies (Figure 5A). The unbound material was then immunoprecipitated with acetyl-H3 antibodies, which showed a marked reduction in c-*fos* and c-*jun* fragments (Figure 5A, lanes 9–12) compared to similar anti-acetyl-H3 IPs from total chromatin (lanes 5–8). This indicates that trimethyl-K4 H3 antibodies had sequestered acetylated H3 in the first round of ChIP. To prove this beyond doubt, anti-trimethyl-K4-immunoprecipitated material was subject to a second round of IP using anti-acetyl-H3 antibody (Figure 5A, lanes 13 and 14). This confirmed that anti-trimethyl-K4 antibody did indeed recover acetylated nucleosomes at these positions on c-*fos* and c-*jun,* accounting for its loss from the unbound fraction. This verifies Western blotting evidence that both modifications are on the same tails and proves the case at c-*fos* and c-*jun*.

Finally, to prove all three modifications occur on the same nucleosomes at c-*fos* and c-*jun,* unbound and bound fractions from control and TPA-stimulated cells, both pre-treated with TSA, were analysed using anti-phosphoacetyl and anti-trimethyl-K4 antibodies (Figure 5B). Cross-linked chromatin was subject to a first IP with anti-trimethyl-K4, and the unbound fraction was analysed in a second IP using anti-phosphoacetyl-H3. Compared to a direct IP using anti-phosphoacetyl antibody, analysis of the unbound fraction after IP with anti-trimethyl-K4 clearly showed depletion of phosphoacetyl epitope (Figure 5B, compare lanes 3 and 4 with lanes 5 and 6). Anti-phosphoacetyl antibody bound material was then subjected to a second IP with anti-trimethyl-K4 antibody (Figure 5B, lanes 9 and 10). This showed that anti-trimethyl-K4 is able to recover chromatin first immunoprecipitated with anti-phosphoacetyl-H3 antibody, indicating that all three modifications are on the same nucleosomes. Taken together, these studies prove that all three modifications, S10 phosphorylation, K9 acetylation, and K4 trimethylation, can certainly co-exist on the same nucleosome, possibly on the same H3 tail, at transcriptionally active c-*fos* and c-*jun*.

Sequential IP experiments, together with Western blotting data, prove conclusively that all three modifications can co-exist on the same nucleosome. It is not claimed here that all nucleosomes at these positions along c-*fos* and c-*jun* carry all three modifications, but rather that a sub-fraction of chromatin fragments must carry trimethyl-K4, acetyl-K9, and phospho-S10 to give a triply modified nucleosome. This follows from the previously established highly dynamic nature of acetylation and phosphorylation at these positions [5]. The fraction that is triply modified represents a “snapshot” of a dynamic process frozen at a particular moment by the cross-linking protocol used.

### Effect of TSA Pre-Treatment on c-*fos* and c-*jun* Induction

We next investigated the functional significance of turnover of histone acetylation observed above for transcriptional activation of c-*fos* and c-*jun*. A major theme in the literature, dating from the original discovery of histone acetylation, suggests that enhanced acetylation correlates with more relaxed chromatin and greater transcriptional activity. If true, the expectation is that TSA treatment would lead to enhanced induction of these genes. If, however, it is the dynamic turnover of acetyl groups observed at these nucleosomes that is critical, then inhibition of turnover with TSA would be expected to lead to inhibited gene induction.

**Figure 6:**
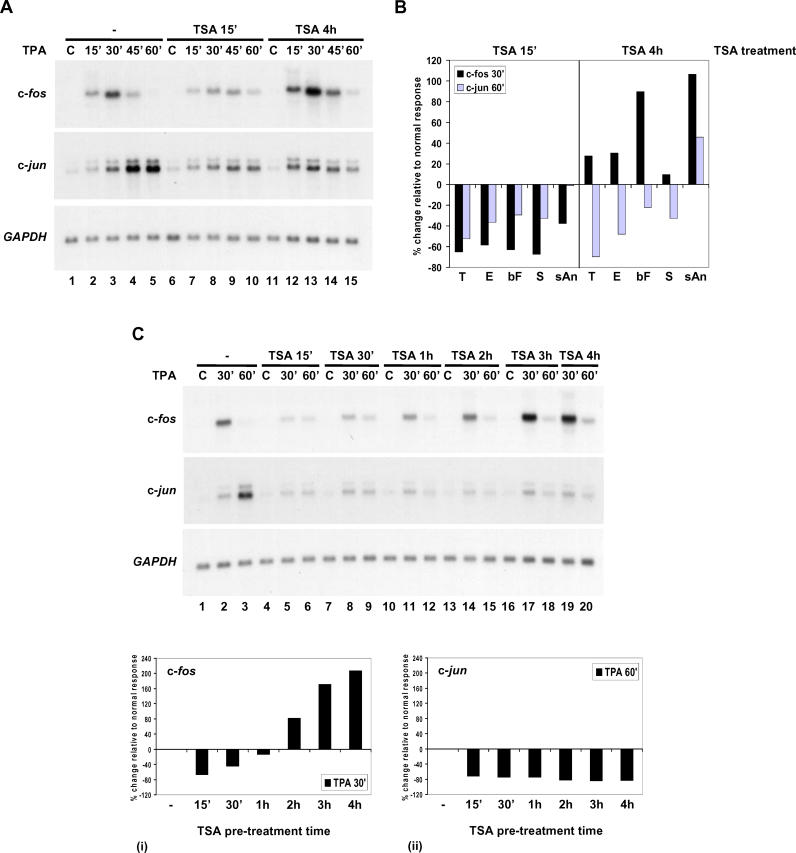
Effect of TSA Pre-Treatment on c-*fos* and c-*jun* Induction (A) Quiescent C3H 10T½ cells were untreated (−) or pre-treated with TSA (500 ng/ml) for 15 min (TSA 15′) or 4 h (TSA 4h). Cells were left unstimulated (C) or stimulated with TPA for 15 to 60 min, and RNA was analysed by Northern blot. (B) Quiescent C3H 10T½ cells were untreated, or pre-treated with TSA (500 ng/ml [TPA- and EGF-stimulated cells] or 10 ng/ml [bFGF-, serum-, sAn-stimulated cells]) for 15 min or 4 h. Cells were left unstimulated, or stimulated with TPA (T), EGF (E), bFGF (bF), serum (S), or sAn for 30 or 60 min. (C) Quiescent C3H 10T½ cells were untreated (−) or pre-treated with TSA (500 ng/ml) for 15 min (TSA 15′), 30 min (TSA 30′), 1 h (TSA 1h), 2 h (TSA 2h), 3 h (TSA 3h), or 4 h (TSA 4h). Cells were left unstimulated (C) or stimulated with TPA for 30 or 60 min. Northern blots in (B) and (C) were quantified by phosphorimaging, corrected for variations in loading using *GAPDH,* and expressed graphically. The normal response at 30 min (c-*fos*)/60 min (c-*jun*) was set to 0%, and inhibition/enhancement in response to TSA pre-treatment expressed as a percentage change relative to this value.

In preliminary studies, we found that TSA itself did not induce these genes, but its effect on c-*fos* or c-*jun* induction by other agents was dependent on the duration of TSA pre-treatment. The phorbol ester TPA produces rapid, transient activation of c-*fos* and c-*jun* in C3H 10T½ cells (Figure 6A; [12]) and Swiss 3T3 cells (Figure S2). In both cell types, short pre-treatment with TSA (15 min; Figure 6A, lanes 6–10; Figure S2, lanes 4–6) inhibited TPA-stimulated induction of both genes. By contrast, longer pre-treatment (4 h; Figure 6A, lanes 11–15; Figure S2, lanes 7–9) produced gene-specific effects, enhancing c-*fos,* but continuing to inhibit c-*jun* induction. We examined the effect of short and long TSA pre-treatment on c-*fos* and c-*jun* induction in response to several other stimuli: EGF, bFGF, serum, and anisomycin (Figure 6B). The inhibitory effect of short (15 min) TSA pre-treatment on induction of these genes was very consistent irrespective of the stimulus. By contrast, its effect after 4 h was both gene- and stimulus-specific (Figure 6B).

Finally, we performed detailed time-course analyses (15 min to 4 h) of TSA pre-treatment to determine precisely when the changeover from inhibition to enhancement of c-*fos* induction occurs (Figure 6C). TPA-stimulated induction of both genes was inhibited by 15 min to 1 h of TSA pre-treatment (Figure 6C, lanes 4–12). The switch from inhibition to enhancement of c-*fos* occurred between 1 and 2 h of TSA pre-treatment (Figure 6C, panel i). Beyond 2 h, TSA clearly enhanced TPA-stimulated c-*fos* expression (Figure 6C, lanes 13–20). By contrast, there was no switch to enhancement with longer TSA treatment for c-*jun,* and similar levels of inhibition were seen over the entire time course (Figure 6C, panel ii). Thus, in the case of c-*fos,* but not c-*jun,* a switch from inhibition to enhancement occurred between 1 and 2 h of TSA pre-treatment. Note that these data confirm that TSA itself does not induce c-*fos* and c-*jun* in these cells (Figure 6C, lanes 4, 7, 10, 13, and 16; Figure 6A, lanes 6 and 11).

In further experiments, we made additional advances on these observations. First, the early inhibitory effect on gene induction was extremely TSA-sensitive and could be observed at a concentration of 1 ng/ml TSA (Figure S3A). Second, inhibition was extremely rapid, and could be observed even if TSA and stimulus were added at the same time or indeed for up to 5 min after stimulation of these cells (Figure S3B), but interestingly, not 10 min after stimulation. This could possibly be due to the very rapid peak of transcription after stimulation, or to the possibility that once transcriptional induction is fully initiated, TSA can no longer affect the process. Third, we found no effect on mRNA degradation rates under these conditions (Figure S4), indicating that inhibition occurred at the transcriptional level, borne out by preliminary ChIP assays using RNA polymerase II antibodies (data not shown). Finally, we found that the later effects of TSA (i.e., beyond 2 h), which varied depending on the gene and stimulus studied (Figure 6B), were secondary effects dependent on fresh translation (Figure S5), whereas the immediate TSA effects were primary and independent of translation. It is not possible to understand the later effects of TSA without knowledge of why exactly translation is required, but it is clear that they do not correlate with enhanced histone acetylation at these genes.

### Inhibition of c-*fos* and c-*jun* Induction Is a Specific Consequence of HDAC Inhibition by TSA

**Figure 7:**
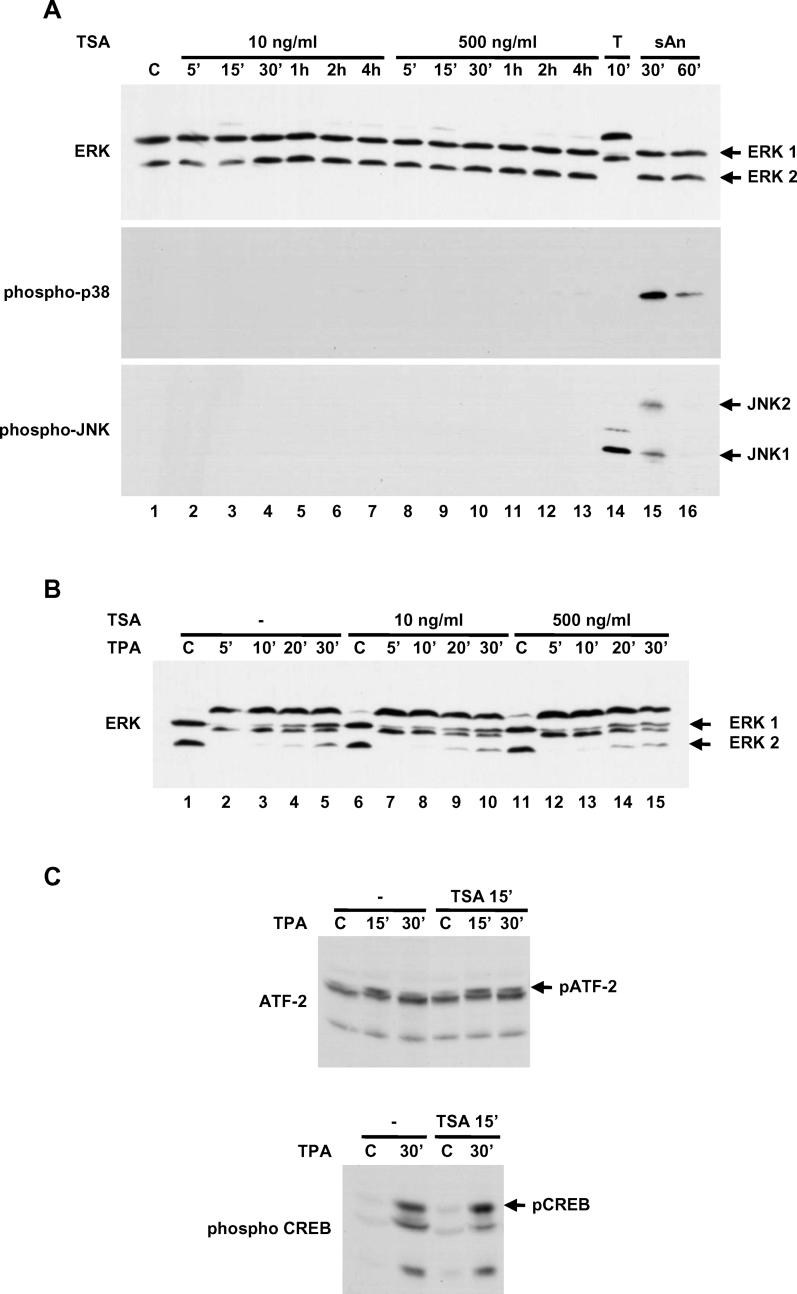
Effect of TSA Pre-Treatment on TPA-Stimulated MAP Kinase Activation and Transcription Factor Phosphorylation (A) Quiescent C3H 10T½ cells were treated with TSA (10 or 500 ng/ml; 5 min to 4 h). Positive controls for MAP kinase activation included ERK1/2 (TPA [T]; 10 min), JNK/SAPKs, and p38 (sAn; 30 to 60 min). “C” indicates control (unstimulated). Cell extracts were analysed by Western blotting with anti-ERK1/2, anti-phospho-p38, and anti–ACTIVE JNK antibodies. The mobility of ERKs is retarded on activation. Activation of p38 and JNK/SAPK results in phosphorylation. Note that anti–ACTIVE JNK also recognises activated ERK1/2 (lane 14). (B) Quiescent C3H 10T½ cells were untreated (−) or pre-treated with TSA (10 or 500 ng/ml; 15 min). Cells were then left unstimulated (C) or stimulated with TPA for 5 to 30 min. Cell extracts were analysed by Western blotting with anti-ERK1/2 antibody. (C) Quiescent C3H 10T½ cells were untreated (−) or pre-treated with TSA (500 ng/ml; 15 min). Cells were stimulated with TPA for 15 to 30 min. Cell extracts were analysed by Western blotting with anti-ATF-2 and anti-phospho-CREB antibodies. Phosphorylation of ATF-2 results in retarded mobility.

**Figure 8:**
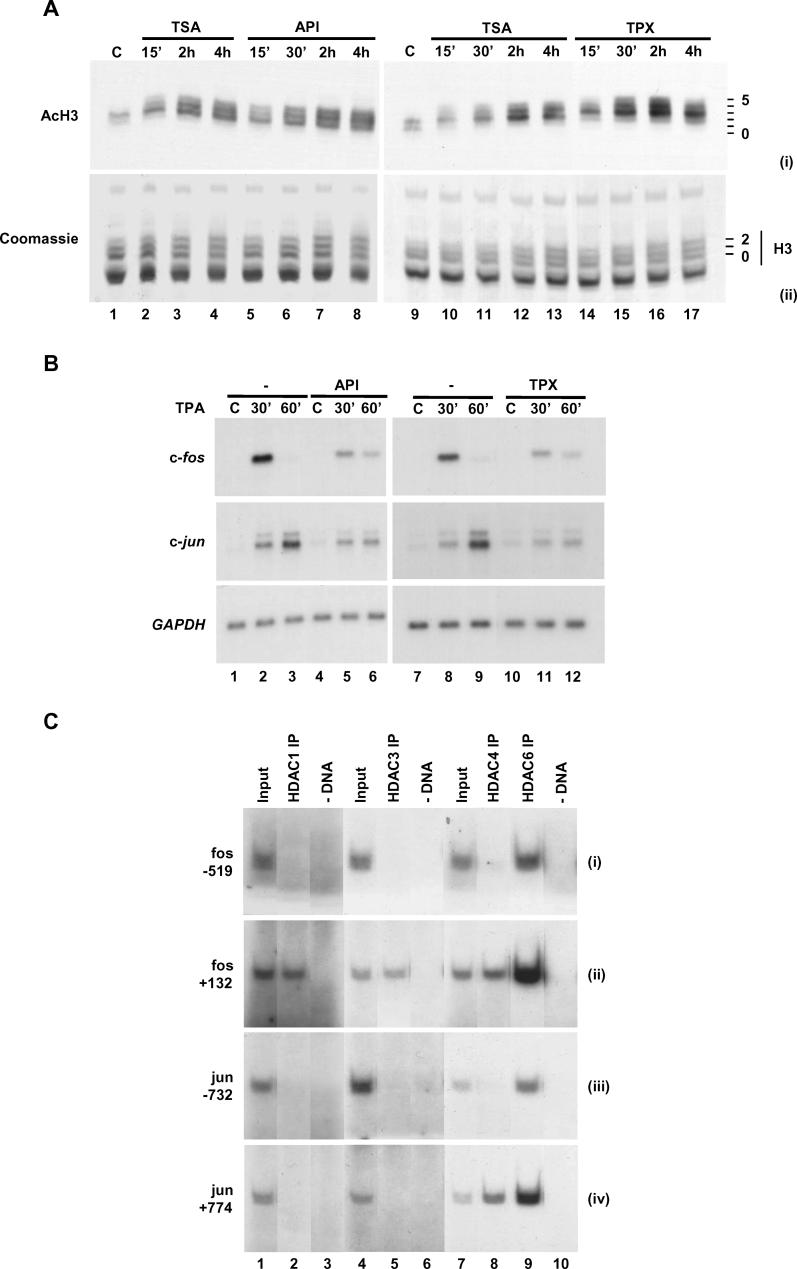
Specificity of Inhibition and Association of HDACs with Regions of c-*fos* and c-*jun* (A) Quiescent C3H 10T½ cells were treated with TSA (10 ng/ml) and HDAC inhibitor API or TPX for 15 min to 4 h. “C” indicates control (unstimulated). Acid-soluble proteins were extracted and separated on acid-urea gels. Western blots were carried out with anti-acetyl-H3 antibodies (panel i). A representative gel was stained with Coomassie to indicate protein loading (panel ii). Positions of histone isoforms are shown on the right of each panel, with zero being unmodified histone H3. (B) Quiescent C3H 10T½ cells were untreated (−) or pre-treated with TSA (10 ng/ml) and HDAC inhibitor API or TPX for 15 min. Cells were then left unstimulated (C) or stimulated with TPA for 30 or 60 min. RNA was analysed by Northern blot. (C) Cross-linked chromatin fragments were prepared from untreated quiescent C3H 10T½ cells. Specific DNAs were immunoprecipitated with anti-HDAC1, anti-HDAC3, anti-HDAC4, or anti-HDAC6 antibodies. Recovered DNAs from antibody-bound fractions as well as total input DNA (Input) from released chromatin used for ChIP were analysed for the presence of c-*fos* (panels i and ii) and c-*jun* (panels iii and iv) gene sequences.

To confirm that the effects of TSA were directly due to inhibition of HDACs and not due to non-specific effects of these compounds, two approaches were taken: first, to verify that TSA does not affect intracellular signalling (Figure 7), and, second, to ask if other HDAC inhibitors produce the same effects (Figures 8A, 8B, and S6). The c-*fos* and c-*jun* genes are critically dependent on MAP kinase cascades and transcription factor phosphorylation for their activation. TSA alone at 10 or 500 ng/ml did not activate any of these MAP kinase cascades (see Figure 7A, lanes 2–13). By contrast, TPA activated ERKs and sub-inhibitory anisomycin (sAn) activated JNKs and p38 (Figure 7A, lanes 14–16). Further, TSA at 10 or 500 ng/ml had no effect on TPA-induced activation of the ERKs (Figure 7B), indicating that it does not affect any of the several signalling steps upstream of ERKs. More critically, we analysed TPA-stimulated phosphorylation of transcription factors ATF-2 and CREB downstream of these cascades and also found no effect of TSA (Figure 7C). Thus, the inhibitory effect of TSA is unlikely to arise from any interference with signalling to transcription factors.

As a second test that inhibition of gene induction is a direct and specific result of HDAC inhibition, we tested three different HDAC inhibitors that are structurally related to TSA (MS-275, CBHA, and M344; Figure S6) and two other inhibitors, apicidin (API) and trapoxin (TPX), that are structurally unrelated to TSA and would not be expected to have the same side effects as TSA (Figure 8A and 8B). Preliminary studies determined the concentration of each inhibitor that increased histone H3 acetylation to a level similar to that produced by TSA (data not shown). In every case, at these concentrations, there was a clear correlation between the ability of each HDAC inhibitor to enhance histone acetylation and its ability to inhibit c-*fos* and c-*jun* induction. Treatment with TSA-related inhibitors CBHA and M344 (Figure S6A, panel i, lanes 9–16) and API and TPX (Figure 8A, panel i, lanes 5–8 and 14–17) produced a clear increase in H3 acetylation comparable to that seen in TSA-treated cells, and strongly inhibited TPA-stimulated c-*fos* and c-*jun* induction (Figure S6B, lanes 10–15; Figure 8B, lanes 4–6 and 10–12). By contrast, treatment with MS-275, which produced weak acetylation of histone H3 (Figure S6A, panel i, lanes 5–8) poorly inhibited TPA-stimulated c-*fos* induction, and did not inhibit c-*jun* (Figure S6B, lanes 7–9).

### Differential Association of Specific HDACs with c-*fos* and c-*jun*

The above data allow a hypothesis that HDACs are specifically associated not just with upstream promoters and enhancer elements, but also with the coding regions of these genes even when quiescent, thereby explaining turnover of acetylation at these positions. To test this hypothesis, we used antibodies against specific HDACs (HDAC1 and HDAC3–HDAC7) in ChIP assays using quiescent cells, analysing both promoter and coding regions of these genes. Using these antibodies, the clearest and most reproducible associations with these genes were obtained with anti-HDAC1, −3, −4, and −6 antibodies (Figure 8C; data not shown). HDAC6 was found to be associated with the coding region and promoter of both c-*fos* and c-*jun* (Figure 8C, lane 9). We also observed coding-region-specific association of HDAC4 for both c-*fos* and c-*jun,* but not at the promoters of these genes (Figure 8C, lane 8; data not shown). HDAC1 and HDAC3 were only seen at the c-*fos* coding region and were not detected at c-*jun* (Figure 8C, lanes 2 and 5). This shows that several HDACs can be found specifically associated with particular regions of c-*fos* and c-*jun,* which can account for the continuous turnover of histone acetylation that we report here. Due to the lower recovery of chromatin fragments using these HDAC antibodies, we may only have been able to detect the most abundant HDACs present at specific regions and cannot rule out the presence of other HDACs at these regions.

## Discussion

Turnover of acetyl groups on histone tails [13] has been a contentious issue since the discovery of acetylation (reviewed in [14]). Metabolic labelling of bulk histones suggests at least two populations with very fast (half-life of 1–5 min) and moderately fast (half-life of 30–60 min) turnover rates (reviewed in [14]). ChIP-based analyses of yeast promoters reveal localised transient histone acetylation associated with remodelling [15], as well as rapid targeted reversal of acetylation after removal of either acetylase (deacetylation within 1.5 min) or deacetylase (acetylation restored in 5–8 min) from a promoter [16]. This model of dynamic rather than stable modification in yeast is supported by the association of the HDAC Hos2 with coding regions of active genes [17]. Evidence of very fast turnover of acetyl groups on a sub-fraction of histones in mammalian cells came from metabolic-labelling studies of phosphorylated H3, which rapidly shifts to the highest acetylated forms upon HDAC inhibition [3].

Detailed analysis of the effects of TSA and other HDAC inhibitors on histone H3 acetylation and c-*fos* and c-*jun* induction here has produced two surprising results. First, these analyses show very highly targeted TSA hypersensitivity, uniquely targeted to all phospho-S10 and methyl-K4, but not dimethyl-K9, H3 in the mouse nucleus. These multiply modified TSA-hypersensitive nucleosomes have been localised to specific regions on c-*fos* and c-*jun,* indicating that these genes are subject to continuous acetylation and deacetylation irrespective of transcription. Second, inhibition of deacetylases at these genes rapidly enhances histone acetylation but inhibits transcription; contrary to the predominant view that increased histone acetylation is characteristic of enhanced transcription. Our data suggest an alternative model, whereby turnover of acetyl groups on K4- methylated histone H3 tails is both characteristic of the poised c-*fos* and c-*jun* genes, and is required for their efficient induction.

### Differential Sensitivity of Nuclear Events to HDAC Inhibition

Classification of TSA sensitivity in the mouse nucleus would have TSA-insensitive genes such as β*-globin* (see Figure 4) at one extreme, followed by a broad swathe of TSA-responsive events, including many in the literature for which high levels of TSA and long treatments are required (see Table S1). Within this second category, data presented here raise the serious complication that some TSA-responsive phenomena, e.g., enhanced c-*fos* induction after 4 h of TSA pre-treatment (see Figure 6A), are secondary events requiring fresh translation (see Figure S5), unrelated to the state of acetylation at the gene, which by 4 h has subsided (see Figure 2). At the other extreme, TSA-hypersensitive genes can be defined as those at which the TSA effect is virtually instantaneous, requiring low concentrations of TSA and detectable by immediate localised histone acetylation. This provides both a molecular model of localised cycling acetylation at these genes, as well as an experimental definition for their detection: brief treatment with TSA followed by ChIP using acetyl-specific antibodies recovers chromatin containing these genes. Here, to our knowledge for the first time, we have demonstrated TSA hypersensitivity directed towards all K4-methylated H3 in the mouse nucleus but not K9-methylated H3. Furthermore, K4-methylated TSA-hypersensitive histone H3 occurs at the poised c-*fos* and c-*jun* genes irrespective of transcription, as well as at the continuously transcribed *GAPDH* (polymerase II) and 18S rRNA (polymerase I) genes, but not at a gene transcribed by RNA polymerase III (tRNA) or silenced in these cells (β*-globin;* see Figures 3 and 4; data not shown). This raises the possibility, discussed further below, that K4 methylation acts as a prior mark that renders histone H3 particularly susceptible to HATs and/or TSA-sensitive HDACs. In yeast, support for this model comes from the recent observation that SAGA and SLIK histone acetyltransferase-containing complexes also contain the chromodomain-containing protein Chd1 that binds to K4-methylated histone H3 [18].

Owing to differential sensitivity of individual HDACs to inhibition with TSA ([19]; reviewed in [20]), effects seen at the lowest concentrations used here are likely mediated by HDACs particularly sensitive to TSA, with other HDACs becoming involved at higher concentrations. Furthermore, we do not exclude the possibility that acetylation of non-histone proteins may also contribute to the inhibition of c-*fos* and c-*jun* seen here. We failed to detect such non-histone acetylation events using various anti-acetyllysine antibodies, but this may be because of limitations of reagents or sensitivity of techniques used. However, the highly targeted enhancement of histone acetylation specifically at c-*fos* and c-*jun* offers an obvious and local cause for their inhibited induction. Until any non-histone acetylation events can be demonstrated at these genes, the simplest interpretation is that the proven histone acetylation shown here is responsible for these observations.

### Deployment of Histone-Modifying Enzymes to Produce Dynamic Acetylation of K4-Methylated Histone H3

TSA-hypersensitive chromatin and K4-methylated H3 must be under continuous opposed action of HATs and HDACs implying micro-compartmentalisation of HAT and/or HDAC function in mammalian nuclei. An H3 K4 methyltransferase must also be directed to these same nucleosomes, but because K4 methylation at these genes appears stable in all the experiments described here and over longer time courses through the cell cycle (data not shown), it is not clear when K4 methylation is deposited, an issue currently under investigation. These two targeted modifications, methylation and the turnover of acetylation, are constitutive in quiescent cells and differ from the third modification, phosphorylation, in not requiring activation of signalling cascades or gene induction.

Localised dynamic turnover of acetylation may be explained by physical distribution of HATs and HDACs in three conceivable ways: (1) HATs are tightly restricted while HDACs act in a diffuse global way, (2) HDACs are tightly restricted while HATs act in a diffuse global way, or (3) both enzymes are tightly co-localised to these genes. It is unlikely that HATs act in a diffuse global mode, as there is much evidence that protein–protein interactions with transcription factors target HATs to genes. Local recruitment of HATs such as CREB-binding protein (CBP), p300, and PCAF via interactions with CREB, SRF, and Elk-1 at regulatory elements upstream of c-*fos* [21,22], and with c-Jun, ATF-2, and MEF upstream of c-*jun,* have been described [23,24]. Association of pre-assembled Elk-1/CBP complexes with gene promoters such as c-*fos* in the absence of transcriptional activation has been demonstrated, but CBP activation is reported to require MAP kinase signalling and Elk-1 phosphorylation [21,25]. Our data show that HATs act continuously, irrespective of cell stimulation or transcription at these genes. Furthermore, TSA-induced acetylation occurs not just at promoter nucleosomes but also more distantly in the body of the gene, as far as ten nucleosomes away for c-*jun* and four nucleosomes away for c-*fos,* but not across the entire transcribed region in either case (see Figure 2). Although there are HATs in RNA pol II holoenzyme and elongation complexes [26], turnover of acetylation occurs independently of transcription and, furthermore, also occurs in upstream non-transcribed regions.

Although targeted HATs alone might explain TSA hypersensitivity, it is likely that HDACs are also targeted to these genes. This has been studied in *Saccharomyces cerevisiae* by deleting specific HATs and HDACs and using ChIP assays to analyse effects on histone acetylation either at specific loci or globally using microarrays [27–29]. These results show that both HATs and HDACs are targeted in yeast, and further, that there is “division of labour” among HDACs [28], e.g., HDAC Sir2p at telomeres, Rpd3p at centromeres, Hos1p and Hos3p at ribosomal DNA, and Hos2p at ribosomal protein genes. For c-*fos* in mammalian cells, mSin3A-HDAC co-repressor complex is reported to be recruited via interactions with Elk-1 [30] but this requires ERK activation and is proposed to mediate shut-off after induction.

Co-location of K4 methylation with H3 acetylation might be implied from the association of both with active transcription (reviewed in [1,2]). Some of the most interesting recent work co-locates H3 acetylation directly with K4 methylation at the same regions of the genome and on the same H3 tail, or places the relevant enzymes in the same complexes. Liang et al. [31], using an adaptation of ChIP assays, found H3 acetylation and K4 methylation around the transcription start sites of several active genes in human bladder cancer cells. H3 has been found on active genes in *Drosophila* euchromatin hyperacetylated and hypermethylated on K4 and K27 [32]. Bradbury and colleagues [33,34], using mass spectrometry, showed that H3 tails that are K4-methylated are also likely to be hyperacetylated, an important study that, unlike the majority of ChIP assays that address localisation to specific regions on a gene, definitively places multiple modifications on the same H3 molecule. Relating to enzyme complexes, the tethering of an HDAC Sin3 to a Trithorax-related K4 methyltransferase (Set1/Ash2) is reported to be mediated by host cell factor-1 (HCF-1) in human cells [35]. Finally, the TAC1 chromatin-modifying complex in *Drosophila* contains the K4 methyltransferase Trithorax and the HAT CBP ([36]; reviewed in [37]).

### Gene Induction and Histone Modifications in the Mouse Nucleus

A major advantage of our mouse model system is that c-*fos* and c-*jun* are poised to transcribe and can be readily induced. Using this system, we find no strict correlation between the K4 methylation state and transcriptional activity. Spatially, K4 methylation spans the start site and extends in both directions at active or poised genes; upstream regions that are not transcribed are K4-methylated, whereas regions furthest downstream that can be transcribed are not K4-methylated. Furthermore, K4 methylation is present whether the gene is constitutively active *(GAPDH),* poised to transcribe (c-*fos* and c-*jun* in control cells), or induced to transcribed (in stimulated cells), but is absent in a constitutively silent gene (β*-globin*). This removes any strict link between K4 methylation and the progress of RNA polymerase II across a gene in mouse cells. Instead, what is established are “islands” of K4 methylation spanning the start sites, whose key characteristic is the continuous turnover of acetylation by the action of HATs and HDACs. Induction of c-*fos* and c-*jun* causes transient MAP-kinase-mediated phosphorylation at S10 on these H3 tails as well as a shift in the HAT/HDAC equilibrium to favour acetylation. Importantly, blocking turnover of acetylation results in inhibited c-*fos* and c-*jun* induction. These two changes are transient whereas pre-existing K4 methylation at these positions remains detectable for many hours afterwards. Temporarily, therefore, a proportion of nucleosomes at these positions on c-*fos* and c-*jun* can be formaldehyde-fixed in a state where they simultaneously carry methyl-K4, acetyl-K9, and phospho-S10 on the same H3 tail. One of the key advances of this work is that it removes comprehensively the notion that “active” genes in the mouse nucleus differ in histone modification from “inactive” genes in a stable and definable way, but highlights instead the highly dynamic nature of these modifications and regional variations across genes. This study, in common with a recent high-resolution histone modification mapping study in yeast [38], challenges the idea of a “histone code” differentiating active from inactive genes.

## Materials and Methods

### Cell culture and stimulation

C3H 10T½ and Swiss 3T3 mouse fibroblasts were grown in DMEM with 10% FCS. Confluent cultures were quiesced by incubation for 16–18 h in DMEM containing 0.5% FCS. Pre-treatment refers to addition of TSA at the times indicated prior to stimulation and left on throughout the period of stimulation until harvest. Cells were pre-treated with histone deacetylase inhibitor TSA (1–500 ng/ml [3.3 nM–1.65 μM]; Sigma, St. Louis, Missouri, United States), API (0.5 μM [39]; Alexis Biochemicals, Montreal, Quebec, Canada), or TPX (500 ng/ml [40]; kindly provided by B. M. Turner, University of Birmingham) and then stimulated with TPA (100 nM; Sigma), EGF (50 ng/ml; Promega, Fitchburg, Wisconsin, United States), bFGF (20 ng/ml; Roche, Basel, Switzerland), 15% FCS (Invitrogen, Carlsbad, California, United States), or sAn (25 ng/ml; Sigma).

### Northern blot analysis of RNA

Gene expression was analysed by Northern blotting as described previously [41]. Blots were sequentially hybridised to c-*fos* and c-*jun* probes and then to the loading control *GAPDH*. Northern blots were visualised by autoradiography and quantified using a PhosphorImager (Molecular Dynamics, Sunnyvale, California, United States).

### Analysis of proteins by Western blotting

Extraction of histones, acid-urea polyacrylamide gel electrophoresis, and Western blotting were carried out as described previously [4,42]. Antibodies were used at the following dilutions: anti-phospho-H3 and anti-phosphoacetyl-H3, 1:1,000; anti-acetyl-H3, 1:2,000; anti-monomethyl-K4 H3, 1:500; anti-dimethyl-K4 H3, 1:2,500; anti-trimethyl-K4 H3, 1:2,000; and anti-dimethyl-K9 H3, 1:2,000. Protein extraction and gel electrophoresis for analysis of MAP kinase activation was carried out as described previously [12,43]. Antibody specificity is described in Protocol S1.

### Chromatin immunoprecipitation

ChIP was performed using formaldehyde cross-linked chromatin as described previously [4] with modifications [5]. ChIPs were carried out using 5 μg of anti-acetyl-H3 antibody/500 μl of chromatin, 25 μg of anti-phosphoacetyl-H3/500 μl of chromatin, and 5 μl of anti-trimethyl-K4 H3/200 μl of chromatin. ChIP analyses using HDAC antibodies were performed using dimethyl adipimidate (Pierce Biotechnology, Rockford, Illinois, United States) and formaldehyde cross-linked chromatin as described in Protocol S1, and 10 μl of anti-HDAC1, −3, −4, or −6/500 μl of chromatin. To analyse unbound chromatin after primary ChIP, unbound supernatant chromatin was removed and retained after incubation with protein A–Sepharose beads (Sigma), and a different second antibody used in ChIP. Sequential ChIPs were performed to analyse histone H3 tails carrying multiple modifications. After primary ChIP, antibody-bound chromatin isolated by binding to protein A–Sepharose was washed and eluted as described previously [4]. Eluted chromatin was diluted to 0.1% SDS, adjusted to RIPA buffer and pre-cleared by incubation with protein A–Sepharose beads for 2 h at 4 °C with rotation. After pre-clearing, the supernatant was concentrated using Vivaspin concentrators (50,000 MWCO; Vivascience, Hannover, Germany). A second ChIP was then carried out using a different antibody. The specificity of antibodies used in ChIPs is detailed in Protocol S1. Quantitative radiolabelled PCR products resolved on 6% polyacrylamide-TAE gels were visualised by autoradiography and quantified by phosphorimaging [4]. Input DNA was diluted one in 60 to approximately 1 ng/μl, and 5 μl was used per PCR reaction. To ensure signals were within the linear range, DNA recovered using anti-acetyl-H3 was diluted one in three and anti-trimethyl-K4 H3 one in 20, and anti-phosphoacetyl-H3 was used undiluted for PCR analyses [5]. DNA recovered using HDAC antibodies was also used undiluted for PCR analyses. Primer names relate to the 5′ position of the forward primer relative to the transcription start site. Details of regions amplified by primers used in this study and sequences of primers are provided in Protocol S1. Similar results were obtained in at least two separate experiments. Quantified PCR data, from PCRs performed in triplicate, are presented as Bound/Input, which corrects for variations in input loading and gives an indication of the relative abundance of immunoprecipitated DNA sequences.

## Supporting Information

Figure S1 Increased Acetylation of Nucleosomes Associated with c-*fos* and c-*jun* Genes on TSA Treatment Chromatin fragments were immunoprecipitated with anti-acetyl-H3 antibodies (see Figure 2). Recovered DNAs from antibody-bound fractions (AcH3 IP) as well as total input DNA (Input) from released chromatin used for ChIP were analysed for the presence of c-*fos* (A) and c-*jun* (B) gene sequences. Quantified PCR data, from PCRs performed in triplicate, are presented here as fold-induction. Note that in contrast to presenting data as Bound/Input (see Figure 2D), which is a measure of the relative abundance of immunoprecipitated DNA sequences, fold-induction shows changes in the level of acetylated H3 on TSA treatment at specific regions of c-*fos* and c-*jun*. Fold-induction appears highest at regions displaying lower basal levels of acetylation. This can be misleading as there can be much greater amounts of acetylation at other regions, as shown by the Bound/Input graphs in Figure 2.
(40 KB PDF).

Figure S2 Effect of TSA Pre-Treatment on c-*fos* and c-*jun* Induction in Swiss 3T3 Cells Quiescent Swiss 3T3 cells were untreated (−) or pre-treated with TSA (500 ng/ml) for 15 min (TSA 15′) or 4 h (TSA 4h). Cells were left unstimulated (C), or stimulated with TPA for 30 or 60 min, and RNA was analysed by Northern blot.
(52 KB PDF).

Figure S3 TPA-Stimulated c-*fos* and c-*jun* Induction Is Hypersensitive to TSA Pre-Treatment (A) Quiescent C3H 10T½ cells were untreated (−) or pre-treated with increasing concentrations of TSA (0.01, 0.1, 1, 10, 100, or 500 ng/ml) for 15 min. Cells were left unstimulated (C) or stimulated with TPA for 30 or 60 min.
(B) Quiescent C3H 10T½ cells were untreated (−) or pre-treated with TSA (10 ng/ml) for 15 min (−15′), 5 min (−5′), or 0 min (0′), and then stimulated with TPA for 30 or 60 min; or cells were first stimulated with TPA, and then TSA (10 ng/ml) was added after 2.5 min (+2.5′), 5 min (+5′), 10 min (+10′), or 15 min (+15′); cells were then harvested after 30 or 60 min of TPA treatment. RNA was analysed by Northern blot.
(59 KB PDF).

Figure S4 Effect of TSA Pre-Treatment on c-*fos* and c-*jun* mRNA Stability Quiescent C3H 10T½ cells were untreated (−) or pre-treated with TSA (500 ng/ml) for 15 min (TSA 15′). Cells were stimulated with TPA (+) for 30 min before the addition of DRB to block transcription. Cells were harvested from 0 to 30 min after DRB addition. As a control for transcriptional inhibition, DRB treatment for 5 min prior to TPA stimulation (30 min) was included. “C” indicates control (unstimulated). Northern blots were quantified by phosphorimaging, corrected for variations in loading using *GAPDH,* and expressed graphically (panels i and ii). The response at TPA 30 min/DRB 0 min was set to 100%, and the rate of mRNA decay expressed relative to this value.
(52 KB PDF).

Figure S5 Effect of the Translational Inhibitor Emetine on TSA-Mediated Changes in TPA-Stimulated c-*fos* and c-*jun* Induction Quiescent C3H 10T½ cells were untreated, or pre-treated with TSA alone (TSA; 500 ng/ml), emetine alone (Em), or emetine plus TSA (Em + TSA) for 15 min or 4 h. Cells were left unstimulated, or stimulated with TPA for 30 or 60 min. Northern blots were quantified by phosphorimaging, corrected for variations in loading using *GAPDH,* and expressed graphically. Values for c-*fos* and c-*jun* mRNA from control untreated cells were negligibly low. The normal TPA response at 30 min (c-*fos*)/60 min (c-*jun*) was set to 0%, and inhibition/enhancement in response to TSA/emetine pre-treatment expressed as a percentage change relative to this value.
(35 KB PDF).

Figure S6 Effect of Different HDAC Inhibitors on Histone H3 Acetylation and c-*fos* and c-*jun* Induction (A) Quiescent C3H 10T½ cells were treated with TSA (10 ng/ml) and HDAC inhibitor MS-275, CBHA, or M344, for 15 min to 4 h. “C” indicates control (unstimulated). Acid-soluble proteins were extracted and separated on acid-urea gels. Western blots were carried out with anti-acetyl-H3 antibodies (panel i). A representative gel was stained with Coomassie to indicate protein loading (panel ii). Positions of histone isoforms are shown on the right of each panel, with zero being unmodified histone H3.
(B) Quiescent C3H 10T½ cells were untreated (−) or pre-treated with TSA (10 ng/ml) and HDAC inhibitor MS-275, CBHA, or M344, for 15 min. Cells were then left unstimulated (C) or stimulated with TPA for 30 or 60 min. RNA was analysed by Northern blot.
(64 KB PDF).

Protocol S1 Additional Experimental Procedures (59 KB DOC).

Table S1 Examples of Different TSA Concentrations and Treatment Times (50 KB DOC).

## Abbreviations

API: apicidin
ChIP: chromatin immunoprecipitation
CBP: CREB-binding protein
*GAPDH*: *glyceraldehyde-3-phosphate dehydrogenase*
HAT: histone acetyltransferase
HDAC: histone deacetylase
IP: immunoprecipitation
K4: lysine 4
K9: lysine 9
S10: serine 10
sAn: sub-inhibitory anisomycin
trimethyl-K4: trimethylated K4
TPA: 12-O-tetradecanoyl phorbol-13-acetate
TPX: trapoxin
TSA: Trichostatin A
