# Supplementary material for: Dynamic Acetylation of All Lysine 4–Methylated Histone H3 in the Mouse Nucleus: Analysis at c-fos and c-jun
Source: PLoS Biol. 2005 Nov 8;3(12):e393. doi: 10.1371/journal.pbio.0030393 (PMC1278937; doi:10.1371/journal.pbio.0030393)
Supplement: Supplementary file 1 [file pbio.0030393.sg001.pdf]

Figure I

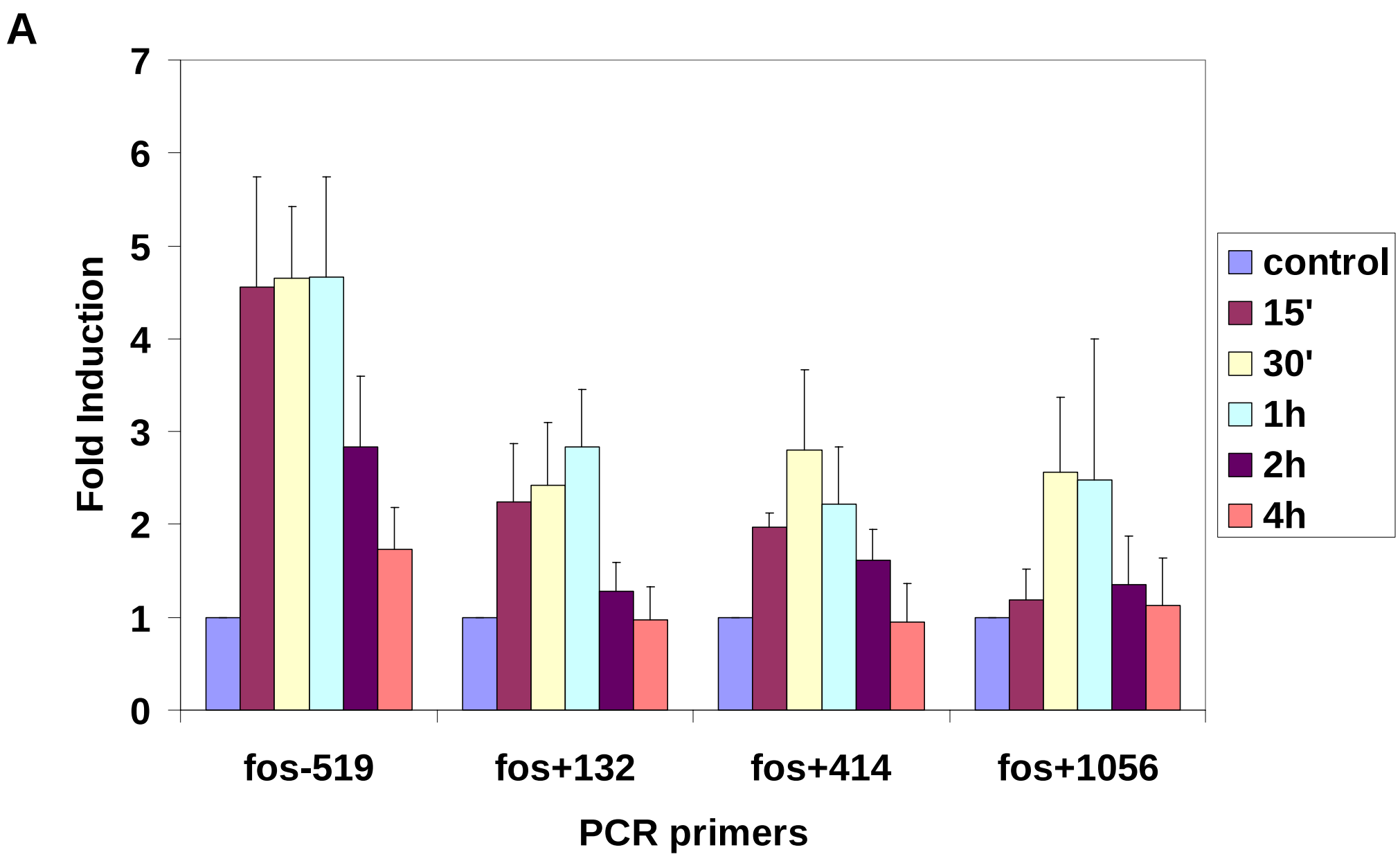
A
Fold Induction
7
6
5
4
3
2
1
0
fos-519
fos+132
fos+414
fos+1056
PCR primers
control
15'
30'
1h
2h
4h

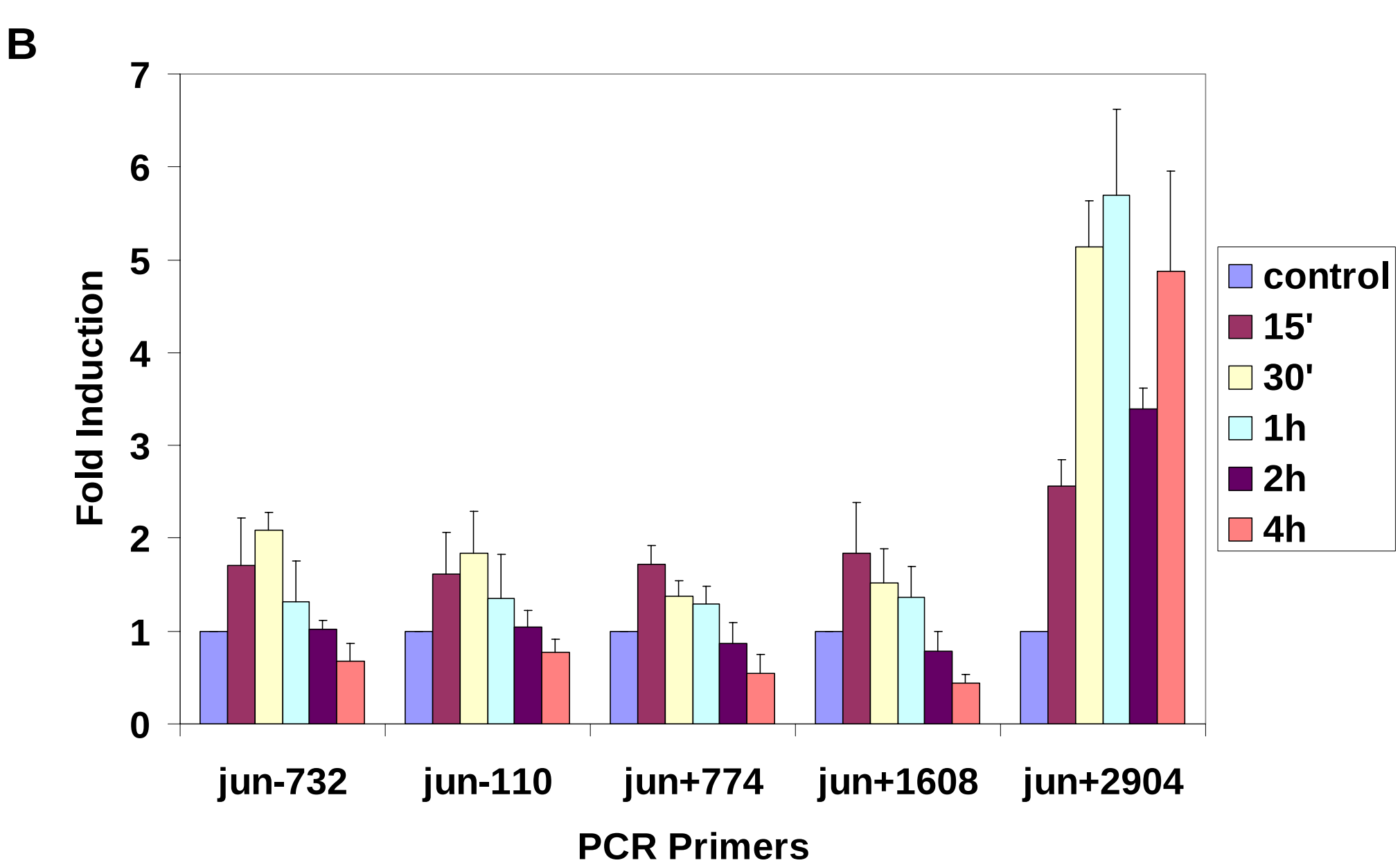
B
Fold Induction
7
6
5
4
3
2
1
0
jun-732
jun-110
jun+774
jun+1608
jun+2904
PCR Primers
control
15'
30'
1h
2h
4h
