# Supplementary material for: Dynamic Acetylation of All Lysine 4–Methylated Histone H3 in the Mouse Nucleus: Analysis at c-fos and c-jun
Source: PLoS Biol. 2005 Nov 8;3(12):e393. doi: 10.1371/journal.pbio.0030393 (PMC1278937; doi:10.1371/journal.pbio.0030393)
Supplement: Supplementary file 2 [file pbio.0030393.sg002.pdf]

Figure II

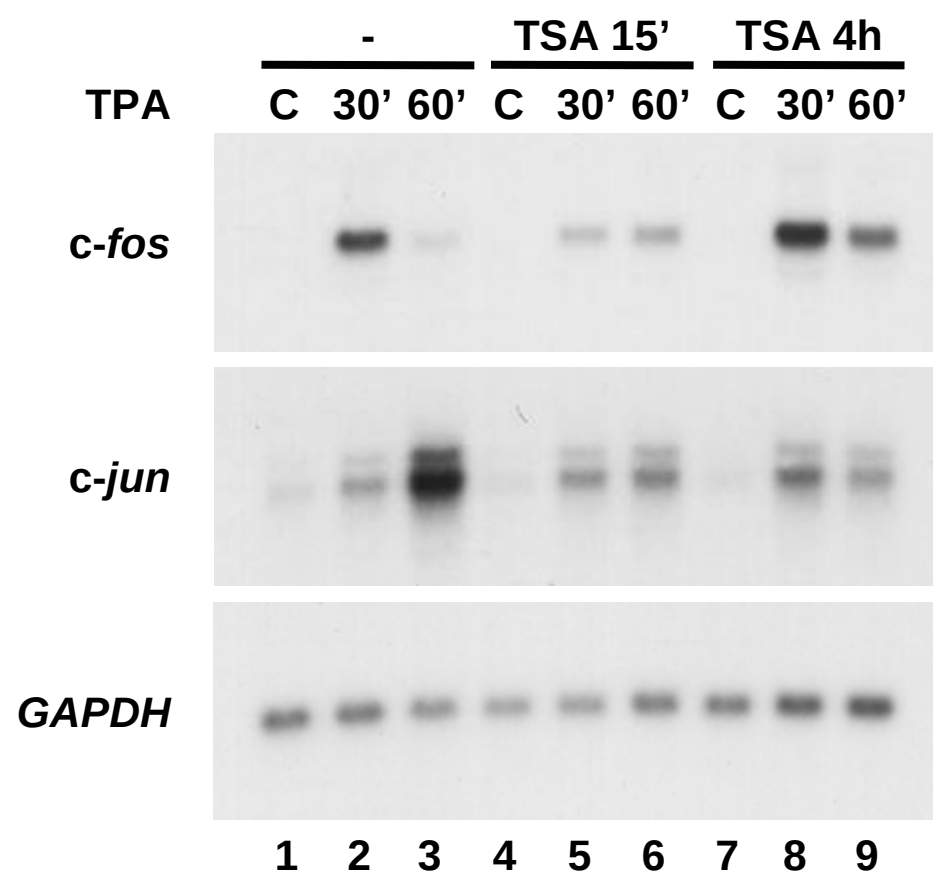
-
TSA 15’
TSA 4h
TPA
C 30’ 60’ C 30’ 60’ C 30’ 60’
c-*fos*
c-*jun*
GAPDH
1 2 3 4 5 6 7 8 9
