# Supplementary material for: Dynamic Acetylation of All Lysine 4–Methylated Histone H3 in the Mouse Nucleus: Analysis at c-fos and c-jun
Source: PLoS Biol. 2005 Nov 8;3(12):e393. doi: 10.1371/journal.pbio.0030393 (PMC1278937; doi:10.1371/journal.pbio.0030393)
Supplement: Supplementary file 4 [file pbio.0030393.sg004.pdf]

Figure IV

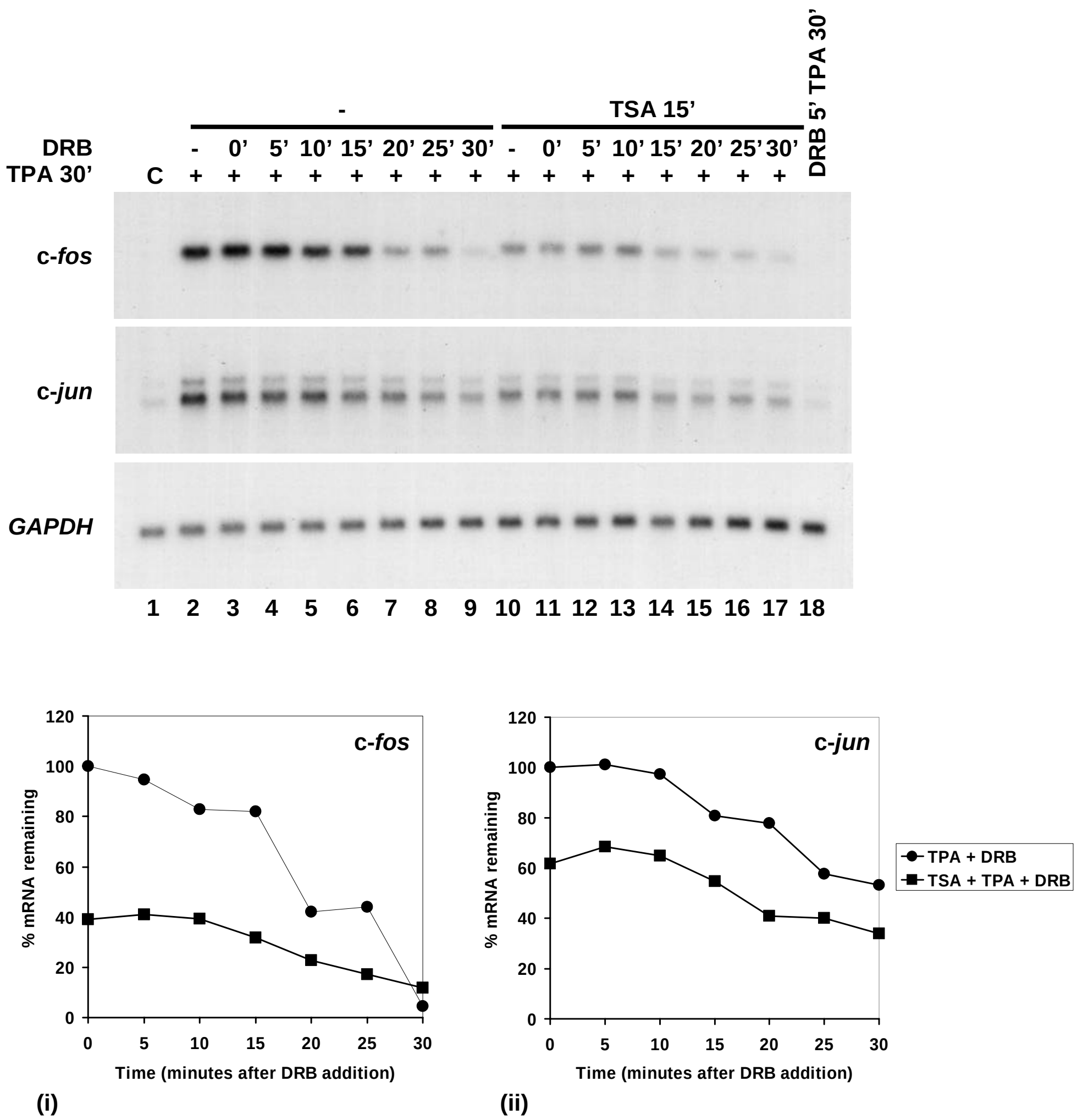

-
TSA 15'
DRB 5' TPA 30'
DRB
TPA 30'
C
- 0' 5' 10' 15' 20' 25' 30' - 0' 5' 10' 15' 20' 25' 30'
+ + + + + + + + + + + + + + + +
c-fos
c-jun
GAPDH
1 2 3 4 5 6 7 8 9 10 11 12 13 14 15 16 17 18
c-fos
% mRNA remaining
120
100
80
60
40
20
0
0 5 10 15 20 25 30
Time (minutes after DRB addition)
(i)
c-jun
% mRNA remaining
120
100
80
60
40
20
0
0 5 10 15 20 25 30
Time (minutes after DRB addition)
(ii)
TPA + DRB
TSA + TPA + DRB
