# Supplementary material for: Dynamic Acetylation of All Lysine 4–Methylated Histone H3 in the Mouse Nucleus: Analysis at c-fos and c-jun
Source: PLoS Biol. 2005 Nov 8;3(12):e393. doi: 10.1371/journal.pbio.0030393 (PMC1278937; doi:10.1371/journal.pbio.0030393)
Supplement: Supplementary file 5 [file pbio.0030393.sg005.pdf]

Figure V

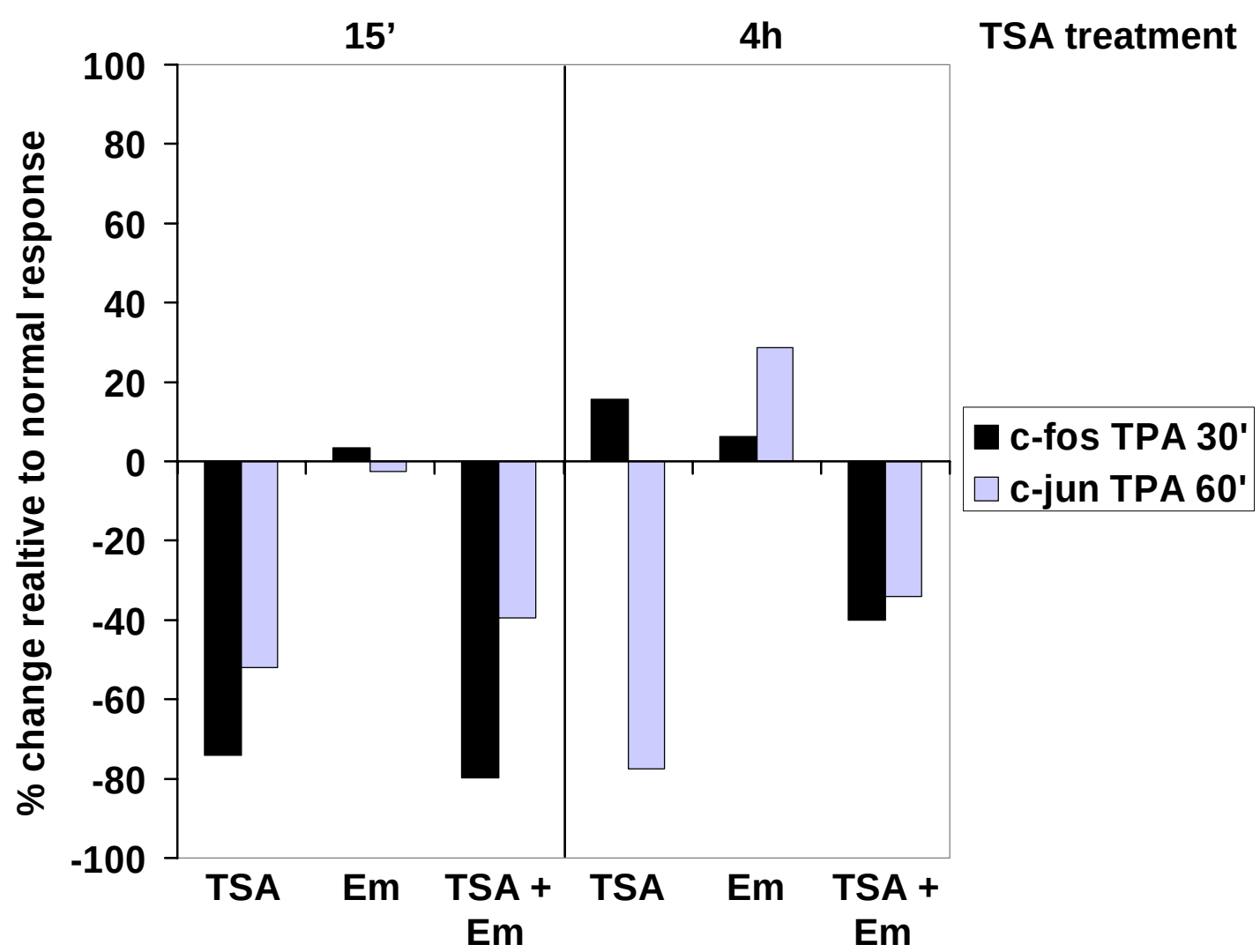

15’
4h
TSA treatment
100
80
60
40
20
0
-20
-40
-60
-80
-100
% change realtive to normal response
c-fos TPA 30'
c-jun TPA 60'
TSA
Em
TSA + Em
TSA
Em
TSA + Em
