# Supplementary material for: Dynamic Acetylation of All Lysine 4–Methylated Histone H3 in the Mouse Nucleus: Analysis at c-fos and c-jun
Source: PLoS Biol. 2005 Nov 8;3(12):e393. doi: 10.1371/journal.pbio.0030393 (PMC1278937; doi:10.1371/journal.pbio.0030393)
Supplement: Supplementary file 6 [file pbio.0030393.sg006.pdf]

Figure VI

A

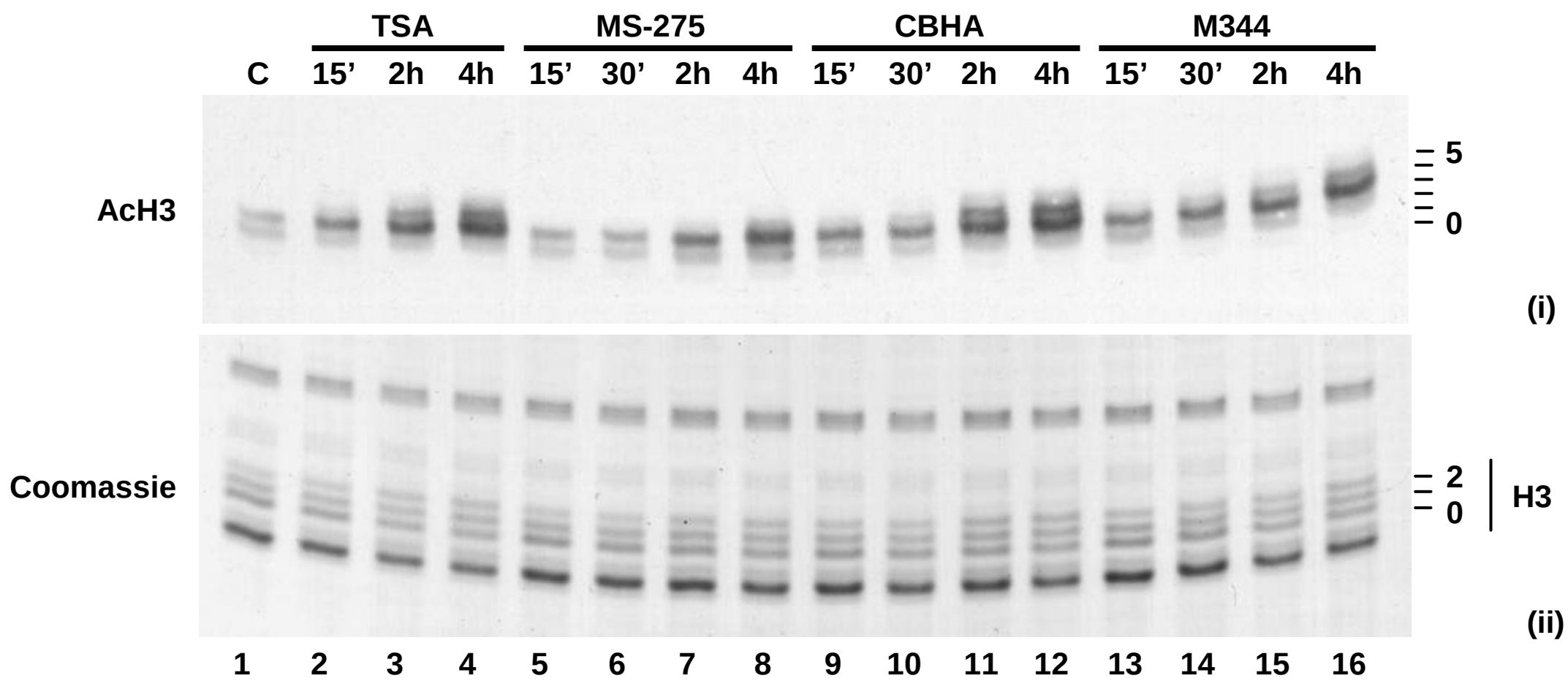
TSA
MS-275
CBHA
M344
C 15' 2h 4h 15' 30' 2h 4h 15' 30' 2h 4h 15' 30' 2h 4h
AcH3
5
0
(i)
Coomassie
2
0
H3
(ii)
1 2 3 4 5 6 7 8 9 10 11 12 13 14 15 16

B

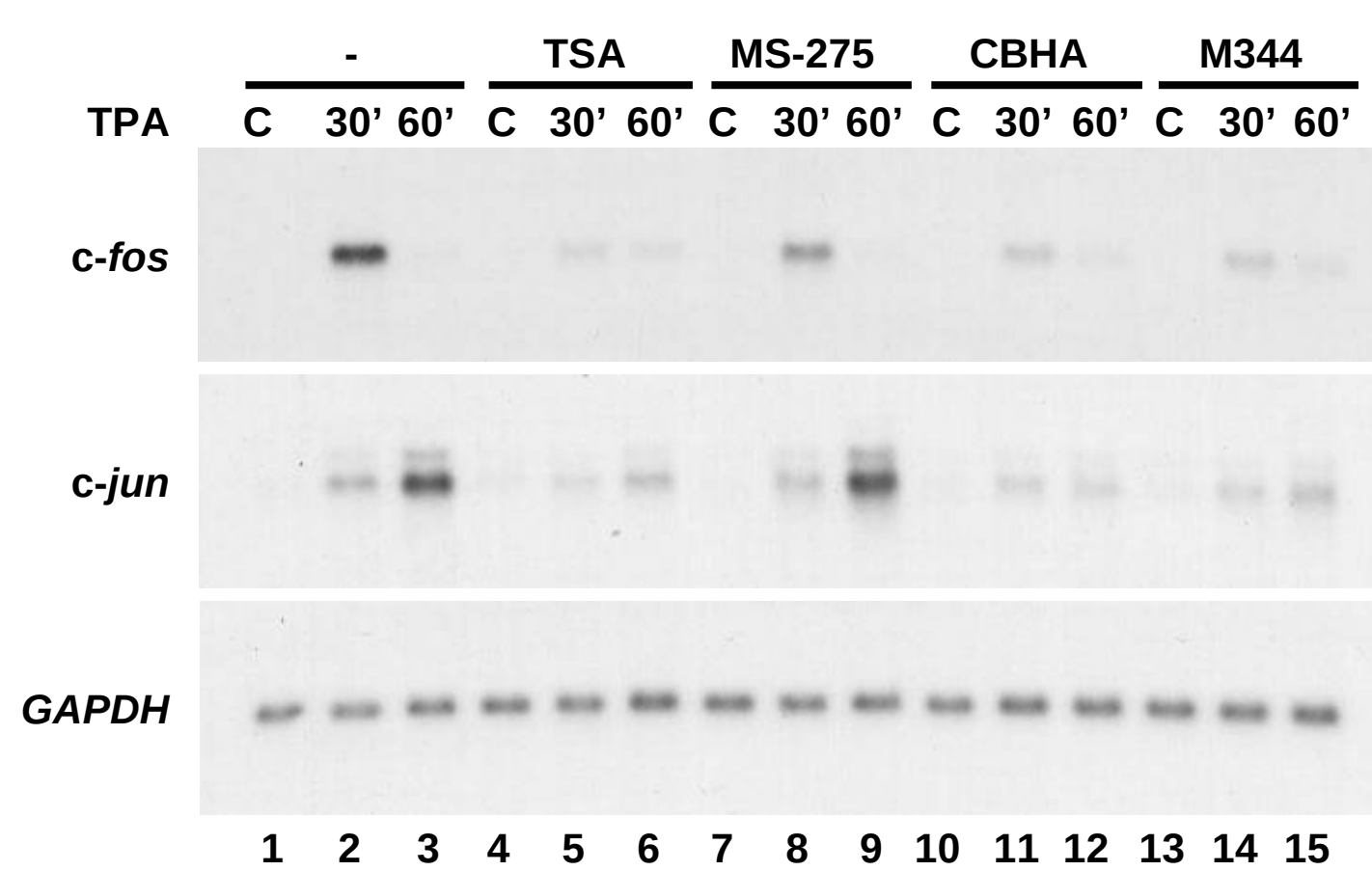
-
TSA
MS-275
CBHA
M344
TPA
C 30' 60' C 30' 60' C 30' 60' C 30' 60' C 30' 60'
c-fos
c-jun
GAPDH
1 2 3 4 5 6 7 8 9 10 11 12 13 14 15
