# Supplementary material for: Dynamic Acetylation of All Lysine 4–Methylated Histone H3 in the Mouse Nucleus: Analysis at c-fos and c-jun
Source: PLoS Biol. 2005 Nov 8;3(12):e393. doi: 10.1371/journal.pbio.0030393 (PMC1278937; doi:10.1371/journal.pbio.0030393)
Supplement: Supplementary file 7 [file pbio.0030393.sd001.doc]

**Experimental Procedures**

**Cell culture and stimulation**

Cells were pre-treated with histone deacetylase inhibitors [MS-275, 10 µM, (Saito et al., 1999); CBHA, 4 µM, (Richon et al., 1998); or M344, 1 µM, (Jung et al., 1999); Calbiochem], and then stimulated with 12-*O*-tetradecanoylphorbol-13-acetate (TPA, 100 nM; Sigma). The protein synthesis inhibitor emetine (Em; Sigma) was used at 10 µg/ml final concentration. The transcriptional inhibitor 5,6-dichlorobenzimidazole 1-β-D-ribofuranoside (DRB; Sigma) was used at a final concentration of 25 μg/ml.

# Antibodies

Anti-phospho H3 antibody (Thomson et al., 1999) recognises histone H3 phospho-Ser10, and H3 phospho-Ser10, acetyl-Lys9, but does not recognise H3 phospho-Ser10, acetyl-Lys14 (Clayton et al., 2000). Anti-phosphoacetyl H3 antibody only recognises doubly modified H3, phospho-Ser10 and acetyl-Lys9. However, acetylation of lysine residues in addition to Lys9 does not inhibit recognition by this antibody (Clayton et al., 2000). Anti-acetyl H3 (Lys9 and 14; Upstate) has high specificity for singly modified acetyl-Lys9 H3 peptides, and does not recognise a peptide acetylated at Lys14 alone (Edmondson et al., 2002). Our studies confirm this antibody strongly binds an acetyl-Lys9 H3 peptide (A. Clayton and L.C.M; unpublished data) but not if Ser10 is also phosphorylated (Thomson et al., 2001). Methylation-specific antibodies anti-monomethyl K4 H3, anti-dimethyl K4 H3, anti-trimethyl K4 H3 (Abcam) and anti-dimethyl K9 H3 (Upstate) are reported to be specific for the mono-, di- and tri-methylated forms respectively, with little or no cross-reactivity to other forms. Antibodies against HDAC1, HDAC3, HDAC4 and HDAC6 were obtained from Cell Signaling Technology.

Anti-ERK1/2 (Zymed Laboratories), anti-phospho p38 MAP kinase that recognises phosphorylated Thr180/Tyr182 (Cell Signaling Technology) and anti-ACTIVE JNK that recognises phosphorylated Thr183/Tyr185 (Promega) were used to analyse MAP kinase activation. Anti-ACTIVE JNK also recognises activated ERK1/2. For transcription factor analyses anti-ATF-2 (Cell Signaling Technology) and anti-phospho-CREB (Upstate) that cross-reacts with p30 and p38 kDa proteins which may be phosphorylated ATF-1 and CREM respectively were used.

# Cross-linked chromatin preparation

To cross-link HDAC proteins which may not be in direct contact with DNA an additional protein-protein cross-linking step was included before formaldehyde treatment to cross-link proteins to DNA. Cross-linking with DMA followed by formaldehyde resulted in more efficient ChIP using HDAC antibodies than formaldehyde cross-linking alone (data not shown). Confluent quiescent plates of cells in culture were treated with fresh DMA (10 mM final concentration) for 10 minutes at room temperature, then formaldehyde cross-linked as described in (Clayton et al., 2000) for a further 10 minutes. Cross-linking was stopped by the addition of glycine to a final concentration of 125mM. Chromatin was prepared as described previously (Clayton et al., 2000) with modifications (Thomson et al., 2001).

# PCR analyses of immunoprecipitated DNA

^32^P-labelled CTP was incorporated into PCR-amplified products for detection and quantification. All PCR reactions were performed using a Perkin-Elmer GeneAmp 2400 thermal cycler and AmpliTaq Gold DNA polymerase. The linear range for all primer pairs was determined empirically using different amounts of C3H 10T½ genomic DNA. Subsequent PCR analyses were carried out using the optimum cycle number determined for each primer pair. The sequences of primers used in this study are as follows: for the c-*fos* gene, fos -519 (amplified region -519→-419, [5’ CCA GAT TGC TGG ACA ATG ACC C 3’, 5’ GGA AAG GCA GAG AAG GCG AGC 3’]), fos+132 (amplified region +132→+318, [5’ GGC TTT CCC CAA ACT TCG ACC 3’, 5’ GGC GGC TAC ACA AAG CCA AAC 3’]), fos+414 (amplified region +414→+624, [5’ GGT CAG AGC AGC CTT AGC CTG 3’, 5’ GGT TTG CCG CCT CCC AAA CTC 3’]), fos+1056 (amplified region +1056→+1242, [5’ CGC AGA TCT GTC CGT CTC TAG 3’, 5’ ACC ATT CCC GCT CTG GCG TAA G 3’]), fos+2622 (amplified region, +2622→+2771, [5’ CTG GTG CAT TAC AGA GAG GAG 3’, 5’ TGG AAG AGG TGA GGA CTG GAG 3’]; for the c-*jun* gene, jun-732 (amplified region -732→-573, [5’ GAG GGC TAC TCT CAA GCC CGC 3’, 5’ GCA CGC CCG AGA AAG GGC TG 3’]), jun-110 (amplified region -110→+114, [5’ CCC CTG AGA ACG ACG CAA GCC 3’, 5’ GAT GAA CAG TCC GGA GTC CGC G 3’]), jun +774 (amplified region +774→+990, [5’ AAG CAC TGC CGT CTG GAG CGC 3’, 5’ TCG GAC TGG AGG AAC GAG GCG 3’]), jun+1608 (amplified region +1608→+1844, [5’ CTG AAG GAA GAG CCG CAG ACC 3’, 5’ GTT CCC TGA GCA TGT TGG CCG 3’]); jun+2904 (amplified region +2904→+3029, [5’ TCA GCC CGC TGG AAA GCA GAC 3’, 5’ CTT CCA TGG GTC CCT GCT TTG 3’]), for the *GAPDH* gene, gapdh-615 (amplified region-615→-490, [5’ CCG CGG GAG GAA AAG TG 3’, 5’ AAA AGG CTG CGG AAA AGT TG 3’]), gapdh+2095 (amplified region +2095→+2181, [5’ GGG CTC ACT ACA CAC CCA TGA 3’, 5’ GTG ACC AGG CGC CCA ATA 3’]), for the *β- globin* gene, globin -841 (amplified region -841→-644, [5’ CAG CAT GTG CTG AGG ACT TGG 3’, 5’ ACT GCC TTC AGA GAA TCG CCC 3’]); globin+261 (amplified region +261→+439, [5’ GCT GCT GGT TGT CTA CCC TTG G 3’, 5’ CAC TGA GGC TGG CAA AGG TG ’]). Primers in the coding regions of the *GAPDH* gene were designed in intron sequences or with one primer in the intron and the reverse primer in exon sequences to prevent amplification of pseudogenes. Sequence positions are given relative to the transcription start site. PCR products were resolved on 6% polyacrylamide-TAE gels, dried and visualised by autoradiography.

# Due to low yields of chromatin following HDAC ChIP, to analyse DNA immunoprecipitated by HDAC antibodies the cycle number was increased to 32 for all primer pairs. Input DNA was diluted to approximately 0.1 ng/µl and 5 µl used per PCR. As a result of the increased PCR cycle number, no statements can be made about the quantitative levels of HDACs associated with specific regions of genes.

**References**

Alberts, A.S., Geneste, O., and Treisman, R. (1998). Activation of SRF-regulated chromosomal templates by Rho-family GTPases requires a signal that also induces H4 hyperacetylation. Cell *92*, 475-487.

Clayton, A.L., Rose, S., Barratt, M.J., and Mahadevan, L.C. (2000). Phosphoacetylation of histone H3 on c-fos- and c-jun-associated nucleosomes upon gene activation. EMBO J. *19*, 3714-3726.

Eberhardy, S.R., D'Cunha, C.A., and Farnham, P.J. (2000). Direct examination of histone acetylation on Myc target genes using chromatin immunoprecipitation. J. Biol. Chem. *275*, 33798-33805.

Edmondson, D.G., Davie, J.K., Zhou, J., Mirnikjoo, B., Tatchell, K., and Dent, S.Y. (2002). Site-specific loss of acetylation upon phosphorylation of histone H3. J. Biol. Chem. *277*, 29496-29502.

Furumai, R., Komatsu, Y., Nishino, N., Khochbin, S., Yoshida, M., and Horinouchi, S. (2001). Potent histone deacetylase inhibitors built from trichostatin A and cyclic tetrapeptide antibiotics including trapoxin. Proc. Natl. Acad. Sci. USA *98*, 87-92.

Jung, M., Brosch, G., Kolle, D., Scherf, H., Gerhauser, C., and Loidl, P. (1999). Amide analogues of trichostatin A as inhibitors of histone deacetylase and inducers of terminal cell differentiation. J. Med. Chem. *42*, 4669-4679.

Li, J., Gorospe, M., Hutter, D., Barnes, J., Keyse, S.M., and Liu, Y. (2001). Transcriptional induction of MKP-1 in response to stress is associated with histone H3 phosphorylation-acetylation. Mol. Cell. Biol. *21*, 8213-8224.

Mariadason, J.M., Corner, G.A., and Augenlicht, L.H. (2000). Genetic reprogramming in pathways of colonic cell maturation induced by short chain fatty acids: comparison with trichostatin A, sulindac, and curcumin and implications for chemoprevention of colon cancer. Cancer Res. *60*, 4561-4572.

Miska, E.A., Karlsson, C., Langley, E., Nielsen, S.J., Pines, J., and Kouzarides, T. (1999). HDAC4 deacetylase associates with and represses the MEF2 transcription factor. EMBO J. *18*, 5099-5107.

O'Neill, L.P., Keohane, A.M., Lavender, J.S., McCabe, V., Heard, E., Avner, P., Brockdorff, N., and Turner, B.M. (1999). A developmental switch in H4 acetylation upstream of Xist plays a role in X chromosome inactivation. EMBO J. *18*, 2897-2907.

Richon, V.M., Emiliani, S., Verdin, E., Webb, Y., Breslow, R., Rifkind, R.A., and Marks, P.A. (1998). A class of hybrid polar inducers of transformed cell differentiation inhibits histone deacetylases. Proc. Natl. Acad. Sci. USA *95*, 3003-3007.

Saito, A., Yamashita, T., Mariko, Y., Nosaka, Y., Tsuchiya, K., Ando, T., Suzuki, T., Tsuruo, T., and Nakanishi, O. (1999). A synthetic inhibitor of histone deacetylase, MS-27-275, with marked in vivo antitumor activity against human tumors. Proc. Natl. Acad. Sci. USA *96*, 4592-4597.

Thomson, S., Clayton, A.L., Hazzalin, C.A., Rose, S., Barratt, M.J., and Mahadevan, L.C. (1999). The nucleosomal response associated with immediate-early gene induction is mediated via alternative MAP kinase cascades: MSK1 as a potential histone H3/HMG-14 kinase. EMBO J. *18*, 4779-4793.

Thomson, S., Clayton, A.L., and Mahadevan, L.C. (2001). Independent dynamic regulation of histone phosphorylation and acetylation during immediate-early gene induction. Mol. Cell *8*, 1231-1241.

Van Lint, C., Emiliani, S., and Verdin, E. (1996). The expression of a small fraction of cellular genes is changed in response to histone hyperacetylation. Gene Expr. *5*, 245-253.

Yang, S.H., Vickers, E., Brehm, A., Kouzarides, T., and Sharrocks, A.D. (2001). Temporal recruitment of the mSin3A-histone deacetylase corepressor complex to the ETS domain transcription factor Elk-1. Mol. Cell. Biol. *21*, 2802-2814.

Yoshida, M., Kijima, M., Akita, M., and Beppu, T. (1990). Potent and specific inhibition of mammalian histone deacetylase both in vivo and in vitro by trichostatin A. J. Biol. Chem. *265*, 17174-17179.
