# Supplementary material for: Dynamic Acetylation of All Lysine 4–Methylated Histone H3 in the Mouse Nucleus: Analysis at c-fos and c-jun
Source: PLoS Biol. 2005 Nov 8;3(12):e393. doi: 10.1371/journal.pbio.0030393 (PMC1278937; doi:10.1371/journal.pbio.0030393)
Supplement: Supplementary file 8 [file pbio.0030393.st001.doc]

| **TSA concentration** | **Treatment time** | **Reference** |
| --- | --- | --- |
| 10 nM | 1–4 h | [8] |
| 330 nM–3.3 µM | 16–20 h | [9] |
| 1 nM–10 µM | 6–24 h | [10] |
| 1.65 µM | 0.5–6 h | [11] |
| 1 µM | 0.5–48 h | [12] |
| 330 nM | 24 h | [13] |
| 16.5–330 nM | 6 h–6 days | [14] |
| 0.1 nM–1 µM | 2–32 h | [15] |
| 330 nM | 18 h | [16] |
| 6.6–330 nM | 6 h | [17] |

**Table I. Examples of different TSA concentration and treatment times**
